# Two-component latency distributions indicate two-step vesicular release at simple glutamatergic synapses

**DOI:** 10.1038/s41467-018-06336-5

**Published:** 2018-09-26

**Authors:** Takafumi Miki, Yukihiro Nakamura, Gerardo Malagon, Erwin Neher, Alain Marty

**Affiliations:** 1 Laboratory of Brain Physiology, CNRS UMR 8118, Paris Descartes University, 45 rue des Saints Pères, 75006 Paris, France; 2 Graduate School of Brain Science, Doshisha University, 1-3 Tatara Miyakodani, Kyotanabe-shi Kyoto, 610-0394 Japan; 3 Department of Pharmacology, Jikei University School of Medicine, Nishi-Shinbashi, Minato-ku Tokyo, 105-8461 Japan; 4 Max Planck Institute for Biophysical Chemistry, Am Fassberg 11, 37077 Göttingen, Germany

## Abstract

It is often assumed that only stably docked synaptic vesicles can fuse following presynaptic action potential stimulation. However, during action potential trains docking sites are increasingly depleted, raising the question of the source of synaptic vesicles during sustained release. We have recently developed methods to reliably measure release latencies during high frequency trains at single synapses between parallel fibers and molecular layer interneurons. The latency distribution exhibits a single fast component at train onset but contains both a fast and a slow component later in the train. The contribution of the slow component increases with stimulation frequency and with release probability and decreases when blocking the docking step with latrunculin. These results suggest that the slow component reflects sequential docking and release in immediate succession. The transition from fast to slow component, as well as a later transition to asynchronous release, appear as successive adaptations of the synapse to maintain fidelity at the expense of time accuracy.

## Abstract (web summary)

Traditionally, synaptic release latency has been viewed as constant. Here, at parallel fiber-interneuron  synapses, authors see that with sustained release emerges a slow component of increased latencies, which can be simulated with a 2-step model comprised of vesicle docking followed by release.

## Introduction

Following action potential (AP) stimulation, the release of synaptic vesicles (SVs) occurs with a jitter that was estimated around 1 ms at the frog neuromuscular junction at room temperature^1^. This jitter reflects the short period of time when the presynaptic calcium concentration ([Ca^2+^]) transient in the vicinity of SVs is large enough to elicit release, as well as the subsequent fusion steps^2–4^, and potentially contains key information on synaptic function. To make precise estimates of the distribution of SV release latencies, deconvolution methods have been applied, taking the mean miniature current waveform as kernel^5^. This method showed that the latency distribution displays a single narrow peak, with a half amplitude duration near 400 μs at room temperature at the calyx of Held synapse^6^.

Apart from a scaling factor according to release probability or to previous synaptic activity, synaptic latencies have classically been considered constant^7,8^. Accordingly, only modest changes of latency distributions have been reported in the calyx of Held^6,9^ and endbulb of Held^10^ during train stimulations. However, central synapses display marked differences in performance during train stimulations^11^. Many central synapses respond to AP bursts at high frequency^12–14^, raising the question of how the potentially conflicting demands of time accuracy and fidelity are reflected at the level of latency distributions during a train.

The classical view that the shape of latency distributions is nearly constant applies if dissociation of Ca^2+^ from release-inducing reaction sites is fast and if the time course of local [Ca^2+^] transients at release sites is constant^15^. On the other hand, long identified reasons to deviate from this scheme include frequency-dependent AP broadening during trains (review: ref. ^16^) and broadening of local [Ca^2+^] following accumulation of Ca^2+^ and/or saturation of fast endogenous buffers (review: ref. ^17^). Another potentially relevant factor is the relation between release kinetics and the distance between SV docking site (DS) and voltage-gated Ca^2+^ channels (VGCCs)^18,19^. If this distance varies among docked SVs, latencies gradually grow during trains due to an increasing participation of low release probability, slow SVs^20^ (review: ref. ^21^). In addition, recent evidence from statistical analysis of release^22^ and flash-and-freeze experiments^23^ indicates ultrafast SV recruitment to DSs, occurring within <10 ms. Such ultrafast docking may elicit a distinct component of release latencies depending on the proportion of docked and undocked SVs. Yet another possible mechanism for latency changes is a slowing of the release step following alterations of the presynaptic structure associated with previous synaptic release^24^ (review: ref. ^25^).

A well-documented phenomenon involving a strong change in synaptic latency is ‘delayed release’, also called ‘asynchronous release’, where release extends for 10 s of ms to 10 s of s following the end of an AP train^26^. Asynchronous release, like synaptic facilitation, depends on the accumulation of residual Ca^2+^ during the AP train, and the two processes may share a common underlying mechanism^27^. In spite of these similarities between facilitation and asynchronous release, it has been suggested that facilitation and asynchronous release each rely on a specific Ca^2+^ sensor (e.g., ref. ^28^), but so far no consensus has emerged concerning the molecular nature of these potential sensors^26^.

In the present work, we investigate the timing of SV release at individual small synapses. During high frequency trains, we find a broadening of release latencies that is much more marked than previously reported at giant synapses, particularly under high release probability conditions. We suggest that latency slowing is due to the insertion of an ultrafast docking step before exocytosis. More generally, the results support a model of SV release involving the sequential transition of SVs to two consecutive states before exocytosis (2-step model: refs. ^22,29,30^). In this model, latency broadening, synaptic facilitation, and asynchronous release rely on a unique sequence of SV docking and release.

## Results

### Response to a single AP

**Fig. 1:**
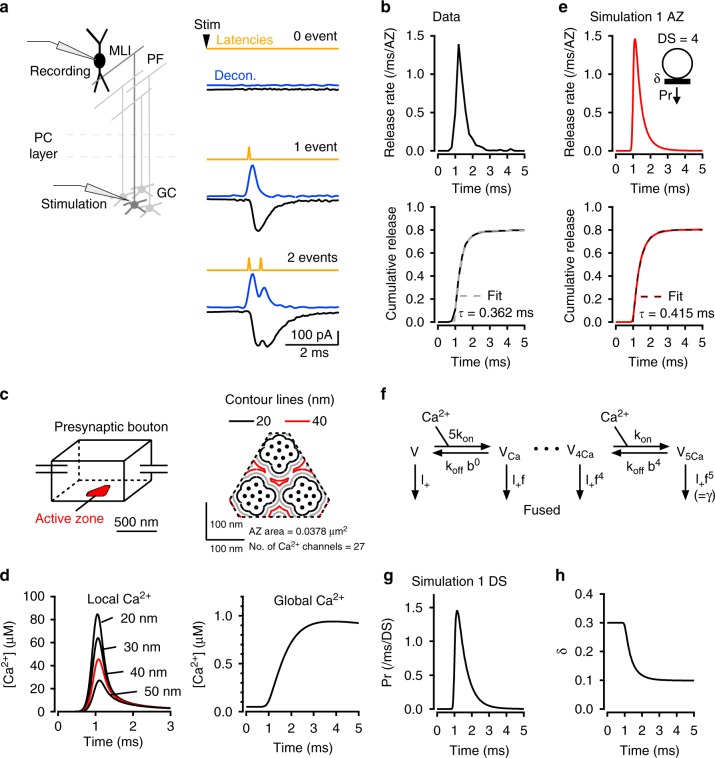
Fast latency distribution following single AP stimulation. **a** Schematics of recording conditions. Representative recordings from a simple PF-MLI synapse, showing individual EPSC responses (black) to presynaptic AP stimuli (stim), deconvolved traces using quantal EPSC as kernel (blue), and calculated latencies obtained by analysis of deconvolved traces (yellow). **b** Upper panel: Release rate for individual simple synapses (average from n = 17 synapses). Lower panel: Corresponding cumulative histogram, with superimposed exponential fit. **c** Schematic representation of presynaptic PF bouton, as used for the calculation of [Ca^2+^] profiles. The AZ has a roughly triangular shape and contains three clusters of VGCCs. SV release occurs near contour lines at a 40 nm distance from VGCC cluster edges (red). **d** Calculation of [Ca^2+^] profiles averaged over the contour lines shown in **c** (left; max. [Ca^2+^] = 45.6 μM at 40 nm distance shown in red), as well as in more distant locations (>100 nm: right; max. [Ca^2+^] = 0.9 μM). **e** Based on the local [Ca^2+^] profile at 40 nm distance, the calculated release rate per simple synapse (with 4 docking sites, an initial docking site occupancy of 0.3, and no replenishment) peaks near 1.5 ms^−1^ (or 1500 s^−1^; upper panel), with a cumulated release probability near 0.8 (lower panel). **f** Allosteric model used for estimation of release rate with following parameters: K_on_ = 5 × 10^8^ M^−1^ s^−1^, K_off_ = 5000 s^−1^, b = 0.75, γ = 2100 s^−1^, f = 31.3 (see Methods). **g** Calculated release rate per occupied docking site, based on global [Ca^2+^] profile in **d** and on allosteric model in **f**. **h** The simulated time course of docking site occupancy, starting from the resting value δ = 0.3. Here no SV replenishment is assumed, so that δ reaches a steady-state value (near 0.1). More complete simulations suggest that δ rebounds after a few ms as shown below

Until now, data on SV release latencies have been only obtained at multisite synapses. In the present work, we analyse the latency distribution of SV release at individual synapses formed between presynaptic parallel fibres (PFs) and postsynaptic molecular layer interneurons (MLIs) in cerebellar brain slices (Fig. 1a). When stimulating a single presynaptic PF, excitatory postsynaptic currents (EPSCs) recorded in MLIs display multivesicular release and EPSC amplitude occlusion at short interevent intervals, as near-simultaneous release from several DSs activates a common set of postsynaptic receptors. These signals arise from SVs released from a single presynaptic active zone (AZ) (‘simple synapse’ recording: ref. ^31^). Variance-mean analysis has shown that each simple synapse has 2–8 DSs^31^. Using deconvolution, we determined the latency of individual SV release events^31^ (Fig. 1a; in this plot, latency is defined as the time difference between presynaptic stimulation and the onset of individual quantal EPSCs).

In response to a single AP in 3 mM external [Ca^2+^], the latency distribution exhibits a rapid rise for about 200 μs, followed by an exponential decay with a time constant of 362 ± 10 μs (Fig. 1b; see fit to cumulative plot in lower panel; note that this latency histogram includes all latencies and not only the first latency in each trial). Because the data originate from single AZs, this latency distribution can be scaled to give the time course of the release rate in one AZ (Fig. 1b). The peak release rate per AZ, ∼1.5 SVs ms^−1^, corresponds to ∼1500 SVs s^−1^, similar to that estimated for a single AZ at the calyx of Held at 2 mM external [Ca^2+^] and at room temperature^6^. Cumulative release amounts to 0.8 SV per AP per AZ (Fig. 1b, lower panel).

The latency as depicted in Fig. 1 comprises many cellular steps, including activation of a presynaptic AP, Ca^2+^ entry following activation of presynaptic VGCCs, intracellular [Ca^2+^] rise near exocytosis [Ca^2+^] sensors, exocytosis, diffusion of glutamate in the synaptic cleft and finally activation of postsynaptic AMPA receptors. Only a few of these steps are likely to contribute to the jitter apparent in Fig. 1a. In control experiments using paired recordings from granule cells and MLIs, we found that the jitter of presynaptic APs in granule cell somata was negligible compared to synaptic jitter (Supplementary Fig. 1). The AP propagation time along axons from soma to terminal is very reproducible and unlikely to create jitter^32^. Because the size of single PF-MLI synapses is particularly small^33^, glutamate diffusion is very rapid and is unlikely to significantly contribute to jitter^34^. Finally, because the time course and amplitude of quantal EPSCs are highly reproducible for a given simple synapse^31^, the delay between glutamate rise and EPSC can be considered as constant. This leaves the steps linking presynaptic VGCC activation to exocytosis as the main source of jitter.

To investigate cellular mechanisms underlying jitter, we simulated SV release in single PF-MLI varicosities. These simple synapses involve a single AZ but several DSs^31,35,36^. Electron microscopic data indicate that individual AZs contain several clusters of VGCCs^37,38^. At simple PF-MLI synapses, the numbers of DSs and of VGCC clusters are similar^38^. Based on morphological data and on the distribution of VGCCs^38^ (Supplementary Fig. 2), we developed a model of a simple PF-MLI synapse AZ (Fig. 1c; Supplementary Fig. 2), and we simulated the spatio-temporal profile of [Ca^2+^] following one AP (Fig. 1d). The amount of Ca^2+^ entry was determined using 2-photon Ca^2+^ imaging at single PF varicosities (Supplementary Fig. 3). Latency distributions were best fitted by placing docked SVs at a distance of 40 nm from the edge of VGCC clusters (red contours, Fig. 1c; this value is intermediate between a <30 nm distance previously estimated at PF-Purkinje synapses^39^ and a 100 nm distance estimated at PF-MLI synapses in culture^40^), giving a maximum local [Ca^2+^] slightly >40 μM (Fig. 1d). By comparison with local [Ca^2+^], the global [Ca^2+^] obtained near the centre of a varicosity displayed slower kinetics and a smaller peak amplitude (Fig. 1d, right); nevertheless, in view of the small size of the varicosity, this peak amplitude was significant (0.9 μM). Based on our previous analysis of variance-mean data^31^, we assumed in our simulations a fixed number of 4 DSs per AZ (Fig. 1e). We further modelled SV release as a sequence of binding to a DS with a resting occupation probability of δ and fusion of a docked SV with a release probability P_r_ per docked SV (Fig. 1e, inset; ref. ^31^). We used an allosteric Ca^2+^-sensor model for release^41^ (Fig. 1f) and the local [Ca^2+^] transient at 40 nm (red profile in Fig. 1d) to produce the time course of P_r_ at one DS (Fig. 1g). This data was then used to reproduce the release rate of four independent DSs (Fig. 1e, red curves), observed in the experiments as latency histogram (compare Fig. 1b, e, lower panels). This analysis assumes an initial DS occupancy (δ) of 0.3 in conformity to previous results^22^ and to simulations of train results shown below (see Methods). Figure 1h illustrates the drop of δ resulting from single AP-evoked SV release (from 0.3 to 0.1, reflecting an integrated release probability of 2/3 for a docked SV).

### Responses to AP pairs

**Fig. 2:**
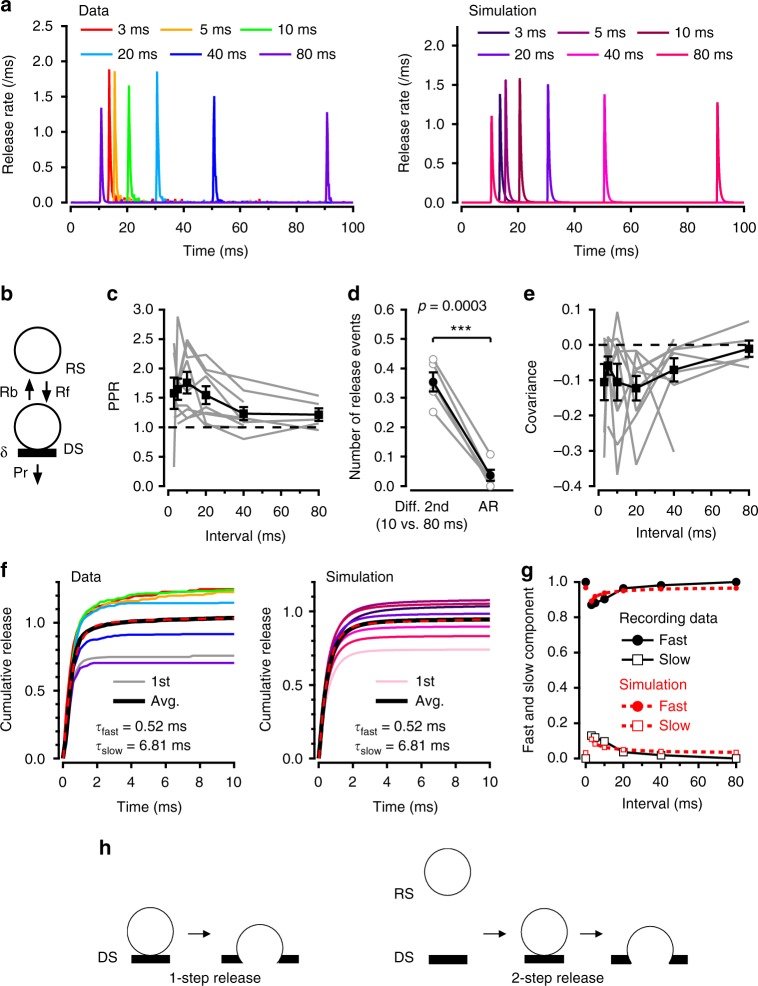
Facilitation and slow latency component in paired stimulation experiments. **a** Pairs of APs with variable inter-AP intervals (from 3 to 80 ms) were applied cyclically (with 10 s intervals between pairs), and release rates were calculated per AZ. Averaging across experiments indicates a transient facilitation peaking near 10 ms and decaying back to control over 80 ms (left). Right: Corresponding simulation. **b** Model of docking/undocking processes for four docking sites per AZ, where each docking site (DS) is associated with a replacement site (RS), with docking rate R_f_ and undocking rate R_b_. **c** Experimental PPR values (where PPR is calculated from the mean numbers s_1_ and s_2_ of released SVs for the two stimuli) for nine experiments (black squares: mean ± sem; grey: individual experiments). **d** Asynchronous release cannot account for facilitation recovery. In each experiment, the difference between mean SV numbers observed after 10 vs. 80 ms was larger than the amount of asynchronous release occurring 10–80 ms after one AP, showing that the recovery of δ cannot be explained on the basis of delayed exocytosis (P_r_ in scheme **b**). Error bars show sem (grey: individual experiments; n = 5). Two-tailed paired *t* test, *t*_(4)_ = 12.1, ***p < 0.001. **e** Covariance between s_1_ and s_2_, as a function of inter-AP interval. **f** Cumulative plots of experimental (left; average of nine experiments) and simulated (right) latencies for first latencies as well as for second latencies with different inter-AP intervals (colour code as in **a**). Note that facilitation is accompanied with the appearance of a secondary slow latency component. Average data of first and all second responses are fitted in both experimental and simulated distributions with the sum of two exponentials with time constants *τ*_fast_ and *τ*_slow_. **g** Plot of *τ*_fast_ and *τ*_slow_ component amplitudes as a function of inter-AP interval (black: experimental; red: simulation). **h** Two different SV release modes. Left: 1-step release shows the classical view of release of a docked SV following AP stimulation. Right: 2-step release shows a rapid sequence of docking (RS → DS) and release following a single AP

We next investigated changes in δ and in release latencies for paired AP stimulations. When stimulating twice, we observed facilitation for inter-AP intervals of 3–20 ms and a return to control amplitudes for longer intervals (Fig. 2a, left). Our previous analysis of SV release statistics suggested a 2-step release model, where SVs transit rapidly from a ‘replacement site’ to an associated DS before exocytosis^22^ (Fig. 2b). Because SV replenishment is Ca^2+^-dependent^42^, the 2-step model assumes that AP trains induce a Ca^2+^-dependent increase in the rate constant of the replacement-docking transition, R_f_^22^ (Fig. 2b; note that this replacement process is not included in the analysis of Fig. 1). This R_f_ increase rapidly reverses the δ drop caused by exocytosis (Fig. 1g). Therefore, depending on the interpulse interval, δ may have fully recovered or even exceeded its basal value when the second AP arrives. For intervals of 10–20 ms, facilitation results at least partially from such an overshoot of δ over its resting value^22^. In this model, the observed dependence of facilitation on inter-AP interval (Fig. 2a, c) implies that δ reaches a peak around 10 ms after the first AP and then decreases back to the resting value in the following ∼100 ms. To explain the return of δ towards resting values, one possibility is a consumption of SVs on DSs due to asynchronous release (P_r_ step in Fig. 2b; here we defined asynchronous release as release events occurring > 5 ms after the preceding AP). At PF-MLI synapses, asynchronous release grows steeply with the number of APs, and both facilitation and asynchronous release depend on a cumulative increase of global [Ca^2+^]^28^. We found that asynchronous release observed after the first AP was too small, by about 1 order of magnitude, to account for the drop of responses between 10 and 80 ms inter-AP intervals (0.036 ± 0.019 per AZ for asynchronous release and 0.354 ± 0.033 per AZ for the difference between 10 and 80 ms; Fig. 2d). This rules out the possibility that asynchronous release is a main cause for facilitation reversal and for δ return and suggests instead the involvement of docking reversibility (transition rate R_b_ in Fig. 2b).

In simple PF-MLI synapses, we observed negative covariance between SV release counts for consecutive APs^22^. This negative covariance reflects the competition between SVs for DSs^43^, so that the covariance time course parallels recovery kinetics of δ following a perturbation. Therefore, if an increase in δ is a major contributor to facilitation (as suggested in Miki et al.^22^), both paired-pulse ratio (PPR) and covariance should follow the recovery of δ as a function of inter-AP interval, displaying the same time course. In accord with this prediction, PPR and covariance displayed similar kinetics (compare Fig. 2c to Fig. 2e), supporting the notion that facilitation follows changes in δ. This does not, however, preclude the alternative or complementary possibility that P_r_ changes contribute to facilitation.

Cumulative latency plots for double stimulations are shown in Fig. 2f, left. Strikingly, while the latency distribution of the responses to the first AP is monoexponential, with a fast time constant (*τ*_fast_ = 0.52 ms), distributions for facilitated responses to the second AP are biexponential, and they display in addition to *τ*_fast_ a slower component with time constant *τ*_slow_ = 6.81 ms (Fig. 2f, left; the first and second responses at different inter-AP intervals are superimposed; same colour code as Fig. 2a). To evaluate the goodness of monoexponential vs. biexponential fits, we used the Bayesian Information Criterion (BIC) that measures the goodness of fit in nonlinear models^44^. For an isolated AP response, the score was smaller for a monoexponential than for a biexponential fit (BIC: −414.4 and −412.9, respectively), indicating that a monoexponential model is better. By contrast, when examining the response to a second AP, we found a substantially better (more negative) score with a biexponential fit (BIC: −576.2) than with a monoexponential fit (BIC: −325.9; analysis performed with 3 ms long interval between the 2 APs). The present finding of a slow latency component for responses to the second AP is consistent with a previous report of a slower average EPSC decay after the second AP than after the first AP^45^. Also in agreement with previous results on EPSC kinetics^45^, the amplitude of the slow component fell off with interstimulus interval in parallel with the extent of facilitation (Fig. 2g, white squares) and the fall in global [Ca^2+^] (Supplementary Fig. 4).

We next asked whether combining the spatiotemporal [Ca^2+^] analysis of Fig. 1 with the 2-step model of Fig. 2b could reproduce the results of paired AP experiments. In these simulations, the local [Ca^2+^] peaked somewhat higher and decayed more slowly for the second AP compared to the first (Supplementary Fig. 4a). In view of the results of Fig. 2d, we included a backward step R_b_ to the model. To account for the Ca^2+^ sensitivity of SV recruitment, R_f_ was assumed to increase with global [Ca^2+^] following a simple binding reaction, as previously proposed^42^ (Supplementary Fig. 4b). Simulations of released SV numbers for the second AP displayed facilitation depending on inter-AP intervals (Fig. 2a, right), due to a transient increase in δ over the basal value (Supplementary Fig. 4c). Remarkably, simulations reproduced the biphasic time course of facilitated release latency distributions, as well as the dependence of both fast and slow component amplitudes on inter-AP interval (Fig. 2f,g). The fast component arises in simulations from SVs that are docked just before the second AP, while the slow component is contributed by SVs that are on a replacement site at the time of arrival of the second AP and that are released in a 2-step process (docking then release, both occurring after the second AP; Fig. 2h). This 2-step process only occurs if R_f_ increases over its resting value in response to a cumulative increase of global [Ca^2+^] during consecutive APs. Thus the decay of the slow component with inter-AP interval follows that of the global [Ca^2+^]. More generally, the simultaneous decay of facilitation, covariance of released SV numbers and *τ*_slow_ component ultimately reflects the common dependence of these phenomena on a transient global [Ca^2+^] increase (Supplementary Fig. 4).

In conclusion, paired AP stimulation experiments reveal a slow release component depending on inter-AP interval and suggest that this component reflects the rapid sequence of docking and release of SVs that were occupying the replacement site before the second AP. Additionally, our results and analyses suggest that the transition between replacement site and DS is reversible.

### Slowing of latency distributions during AP trains

**Fig. 3:**
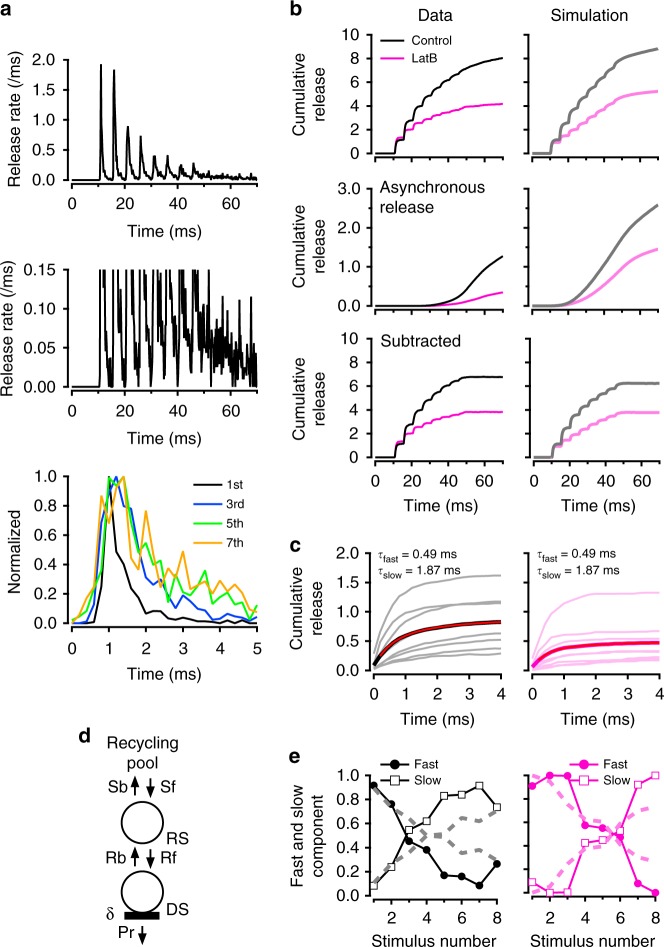
Fast, slow and asynchronous release during trains, in control and in LatB. **a** Average release rate (per ms and per simple synapse; 3 mM external [Ca^2+^] and 200 Hz stimulation; n = 11 experiments). Middle panel: Expanded vertical scale highlighting asynchronous release. Lower panel: Scaled latency distributions for stimuli 1, 3, 5 and 7 are superimposed to show a marked latency broadening during trains. **b** Effect of LatB on cumulative latency counts during trains. Overall latency counts (upper row) have been decomposed into a superslow component representing asynchronous release (middle row) and a phasic component (lower row). Black: Control synapses (3 mM external [Ca^2+^] and 200 Hz stimulation). Red: Synapses pretreated with LatB to inhibit actin-dependent SV movement. **c** Superimposed cumulative latency distributions for each AP stimulation during an 8-AP train, both for control data (left) and in LatB (right). Average traces of 8-AP responses (thick black curve) is fitted with a double exponential (red) with indicated *τ*_fast_ and *τ*_slow_ values. **d** Full model with reversible transition between recycling pool and replacement site (forward: S_f_, backward: S_b_). **e** Relative contributions of *τ*_fast_ and *τ*_slow_ component as a function of stimulus number (symbols and continuous lines: experimental; dashed lines: simulations)

The results so far indicate that double AP stimulation markedly alters latency distributions (Fig. 2). However, while the association of a slow latency component with a second stimulus is clear, the amplitude of this component is modest. To increase the slow component amplitude, we next challenged DS replenishment by applying train stimulations at high frequency, using as before a high reference extracellular [Ca^2+^] (3 mM) to induce high SV turnover (Fig. 3). As expected, a strong synaptic depression was observed during the train, indicating that SV replenishment cannot keep up with release at 200 Hz (Fig. 3a, upper panel). A parallel increase in asynchronous release was apparent at higher magnification (Fig. 3a, middle panel). Here we defined asynchronous release as the envelope of release observed at the end of each inter-AP period, as well as after the end of the AP train (Supplementary Fig. 5). Asynchronous release is thought to correspond to the SV release induced by the increasing global [Ca^2+^] depicted in Fig. 1d, right. After normalization, we found a pronounced widening of latency distributions during trains (Fig. 3a, lower panel). The half maximum width increased from 0.43 ms for the first response to 1.35 ms for the last response. Biexponential fit of mean latency data (black curve, Fig. 3c) gave *τ*_fast_ = 0.49 ± 0.12 ms and *τ*_slow_ = 1.87 ± 0.74 ms.

We next examined how the contributions of the slow release component and of asynchronous release vary in a series of experimental manipulations aiming to challenge the replenishment step and/or to decrease δ. In all cases, latency distributions contained 2 kinetic components, with time constants of ~0.5 and ~2 ms (respective ranges: 0.38–0.59 ms and 1.43–2.93 ms); a satisfactory fit was achieved by keeping common values for *τ*_fast_ and *τ*_slow_, and adjusting the percentage of each component. (Note that the larger value of *τ*_slow_ in Fig. 2 results from a lack of consideration of asynchronous release, as well as from a longer fitting time window, in that figure.).

During trains, δ decreases due to SV depletion at DSs, while the docking rate R_f_ increases due to a cumulative increase in global [Ca^2+^]. If the *τ*_fast_ component arises from docked SVs, its contribution should decrease together with δ. Meanwhile if the *τ*_slow_ component reflects 2-step docking/fusion events, its contribution should increase as R_f_ and free DSs' availability increase. In accordance with these expectations, we found that the amplitudes of *τ*_fast_ and *τ*_slow_ components varied in opposite directions, so that the *τ*_slow_ component exceeded the *τ*_fast_ component near the end of a train (Fig. 3e, left).

Since the data presented so far were collected from young animals (P12‒P16 rats), we considered the possibility that the *τ*_fast_ to *τ*_slow_ transition would be linked to neuronal development. When recording similar data from young adult rats (P26–P29), we also found 2 kinetic components (*τ*_fast_ = 0.54 ms, *τ*_slow_ = 1.32 ms). Amplitudes of fast and slow components varied in opposite directions during AP trains, and the *τ*_slow_ component became dominant at the end of a train (Supplementary Fig. 6). These results suggest that the *τ*_fast_ to *τ*_slow_ transition is maintained during neuronal development.

Next, we assessed the effect of the actin polymerization inhibitor latrunculin B (LatB) that blocks facilitation by inhibiting the docking step R_f_ (ref. ^22^). If the *τ*_slow_ component is due to 2-step release, it should be affected by inhibiting R_f_. In agreement with this prediction, we found that application of LatB (15 μM) resulted in a sharp reduction of the *τ*_slow_ component (Fig. 3b, c, red curves). Consequently, when plotting the percentages of *τ*_fast_ and *τ*_slow_ as a function of stimulus number (*i*), the *τ*_fast_ to *τ*_slow_ crossover occurred later in a train, shifting from *i* = 3 under control conditions to *i* = 6 in LatB (Fig. 3e). Interestingly, asynchronous release, measured during a 60 ms long window starting 5 ms after the end of the train, was inhibited in parallel with the *τ*_slow_ component (control: 1.54 ± 0.39 SV per 60 ms; LatB: 0.47 ± 0.19 SV per 60 ms; mean ± sem; two-tailed *t* test, *t*_(18)_ = 2.46, *p* = 0.027; Fig. 3b, second row). These results suggest that both the *τ*_slow_ component and asynchronous release rely on the docking step R_f_.

**Fig. 4:**
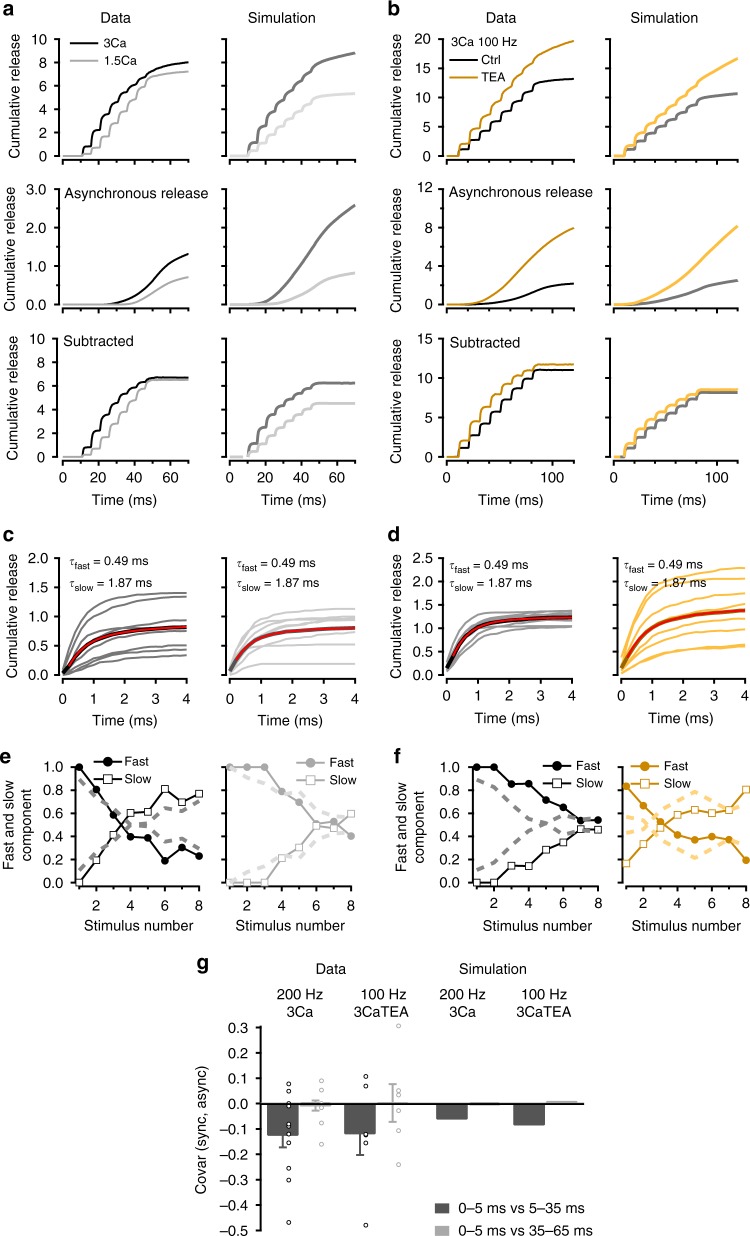
Modifications of release by stimulation frequency, external [Ca^2+^] and TEA. **a**, **b** Effects of various experimental manipulations on cumulative latency counts during trains. Overall latency counts (upper row) have been decomposed into a superslow component representing asynchronous release (middle row) and a phasic component (lower row). **a** Comparison between 1.5 and 3 mM external [Ca^2+^] (200 Hz stimulation in each case). **b** Comparison between control recordings (3 mM external [Ca^2+^] and 100 Hz stimulation) and test recordings obtained after adding 1 mM TEA to increase release probability. **c**, **d** Superimposed cumulative latency distributions for each AP stimulation during an 8-AP train, both for control data (left plots in each condition) and for test data (right plots). Average traces of 8-AP responses (thick black curves) are fitted with a double exponential (red) with the indicated *τ*_fast_ and *τ*_slow_ values. **c** Left: 3 mM external [Ca^2+^], 200 Hz stimulation. **c** Right: 1.5 mM external [Ca^2+^], 200 Hz stimulation. **d** Left: 3 mM external [Ca^2+^], 100 Hz stimulation. **d** Right: As in left, after adding 1 mM TEA. **e**, **f** Relative contributions of *τ*_fast_ and *τ*_slow_ component as a function of stimulus number (symbols and continuous lines: experimental; dashed lines: simulations). **e** 3 mM external [Ca^2+^] (left) vs. 1.5 mM external [Ca^2+^] (right); 200 Hz stimulation in both cases. **f** Control (left) vs. 1 mM TEA (right); 100 Hz stimulation in both cases. **g** Covariance analysis suggests that last phasic SV release shares the same pathway as later asynchronous release (left: experimental; right: simulation). Error bars show ± sem from 11 experiments for 200 Hz 3 Ca and 6 experiments for 100 Hz 3 Ca TEA (open circles: individual experiments)

We next examined the impact of changing the amount of Ca^2+^ entry per AP on *τ*_fast_ and *τ*_slow_ components. If the *τ*_slow_ component is due to 2-step release, it should vary with the amount of Ca^2+^ entry as R_f_ increases with global [Ca^2+^]. In agreement with this prediction, we found that reducing the external [Ca^2+^] from 3 to 1.5 mM decreased the contribution of *τ*_slow_ (Fig. 4a, c). Meanwhile the reduction of the *τ*_fast_ component during the stimulus train was less rapid in the lower external [Ca^2+^] due to a reduction of synaptic depression of this component. Consequently, the *τ*_fast_/*τ*_slow_ crossover shifted again from *i* = 3 to *i* = 6 (Fig. 4e). As expected from previous findings^28^, we also found a dependence of asynchronous release on external [Ca^2+^] (1.5 mM [Ca^2+^]: 0.58 ± 0.04 SV per 60 ms; 3 mM [Ca^2+^]: 1.14 ± 0.22 SV per 60 ms; mean ± sem; two-tailed *t* test, *t*_(10)_ = 2.55, *p* = 0.037; Fig. 4a, middle panels).

If the *τ*_slow_ component relies on Ca^2+^-dependent R_f_ increase, its amplitude should decrease when reducing the stimulation frequency, as the global [Ca^2+^] is then reduced. In agreement with this prediction, reducing the AP frequency from 200 Hz (Fig. 4a; similar group results from another set of data are shown in Fig. 3b, left) to 100 Hz (Fig. 4b) led to a right shift of the crossover, from *i* = 3 to *i* = 7 (Fig. 4e, f, left). To increase Ca^2+^ entry independently of the external [Ca^2+^], we added 1 mM tetraethylammonium (TEA)^46^. With 100 Hz stimulation, the crossover point shifted back to the left, to *i* = 3 (Fig. 4f, right), again as expected. Simultaneously, TEA markedly increased asynchronous release (control: 1.55 ± 0.35 SV per 90 ms; TEA: 6.11 ± 0.77 SV per 90 ms; mean ± sem; two-tailed *t* test, *t*_(10)_ = 5.36, *p* = 0.001; Fig. 4b, second row).

In summary, latency distributions become markedly slower during AP trains, gradually shifting from a process exhibiting a fast time constant (*τ*_fast_ ~0.5 ms) to another one displaying a slow time constant (*τ*_slow_ ~2 ms). Blocking SV docking with LatB, decreasing Ca^2+^ entry and decreasing stimulation frequency all delay the transition from *τ*_fast_ to *τ*_slow_ during an AP train. Conversely, increasing Ca^2+^ influx or increasing stimulation frequency accelerates this transition. Altogether, latency broadening increases with the intensity of previous synaptic activity and with release probability and depends on an intact cytoskeletal structure. All of these changes support the notion that the *τ*_slow_ component results from 2-step release. In addition, asynchronous release follows the same trends as the percentage of *τ*_slow_ component, suggesting that slow release and asynchronous release share a common underlying mechanism.

### Simulation of latency changes during trains

We next asked whether the 2-step model accounts for the results of Figs. 3 and 4. We performed simulations using the 2-step model (Fig. 3d), changing relevant kinetic parameters to mimic the various experimental manipulations. R_f_ was assumed to depend on global [Ca^2+^] as explained above; in addition, its value was reduced during LatB application^22^. Refilling of the replacement pool was modelled assuming a single-step exchange with an infinite SV reservoir (forward and backward rates: S_f_ and S_b_; Fig. 3d). S_b_ was kept constant during a train, while S_f_ increased with global [Ca^2+^] following a simple binding model (Supplementary Fig. 7b). Ca^2+^ entry was adjusted in simulations of experiments changing the external [Ca^2+^] and/or adding TEA according to local [Ca^2+^] measurements performed in individual PF presynaptic terminals (Supplementary Fig. 3, 7 and 8, Supplementary Table 1).

Simulations of local [Ca^2+^] changes during trains suggested that, at 200 Hz and 3 mM external [Ca^2+^], local [Ca^2+^] accumulation is significant, resulting in a widening of P_r_ decay for late AP stimulations and in a slow return to baseline at the end of AP trains (Supplementary Fig. 7a, left and middle columns). This effect was reduced when lowering external [Ca^2+^] (Supplementary Fig. 7c) or stimulation frequency (Supplementary Fig. 8a) and increased by addition of TEA (Supplementary Fig. 8c).

High frequency stimulation leads to substantial incorporation of SV membrane to the AZ. Pending compensatory mechanisms including ultrafast endocytosis^47^, this likely results in the disruption of release machinery, in the accumulation of potentially inhibitory elements left over from SNARE assemblies and from SV proteins, in an increase in the distance between Ca^2+^-sensitive elements and VGCCs and in a reduction of surface tension^24,48–50^. All of these effects are likely to slow the release process (review: ref. ^25^). To model the change of synaptic function following previous synaptic activity, that we call ‘fatigue-induced slowing of release’ below, we altered the parameters describing the release of docked SVs, making the activation steps slower (Supplementary Fig. 7a, right column). Furthermore, we assumed a reset time of 40 ms during which the parameters gradually returned to control values. In LatB simulations, the reset time was increased to 1 s, in line with our previous results^22^.

The simulations shown in Figs. 3 and 4 take into account accumulation of local [Ca^2+^] and fatigue-induced slowing of release. Like 2-step release, both phenomena are expected to gain prominence with AP repetition during trains, as well as with the amount of Ca^2+^ entry. When including P_r_ changes linked to [Ca^2+^] accumulation and to fatigue-induced slowing of release, the model was able to fully account for the slowing of latency distributions in various experimental conditions, as illustrated in Figs. 3 and 4 and Supplementary Fig. 7b‒d and 8b‒d.

### Phasic and asynchronous release share the same pathway

Like facilitation, asynchronous release depends on global [Ca^2+^], but the exact relation between phasic release and asynchronous release remains unclear^28^ (review: ref. ^26^).

Owing to the small size of PF terminals and to the large number of VGCCs present in each AZ, the Ca^2+^ entry associated with one AP results in a sizable global [Ca^2+^] elevation (0.9 μM: Fig. 1d). During AP trains, this global [Ca^2+^] elevation accumulates and reaches several μM^22^ (Supplementary Fig. 3), a level sufficient to elicit significant SV release^41^. Thus the level of [Ca^2+^] at DSs near and after the end of an AP train is sufficient to provide a continuous release flux (Figs. 3b and 4a, b, second row), suggesting that DSs operating for phasic release under standard rules are responsible for asynchronous release. The above finding that LatB inhibits asynchronous release is in line with this hypothesis.

To further test this hypothesis, we compared released SV counts following the last AP of a train of 8 (s_8_, time window: 0–5 ms following the last AP) with asynchronous release counts in the following 30 ms time window (time window: 5–35 ms after the last AP). If phasic and asynchronous release are independent of each other, the covariance between these two counts should be nil. If on the other hand the two types of release compete for a common limited resource, for example by sharing the same release sites, the covariance should be negative^43^. We found a negative covariance between s_8_ and asynchronous release, both in 3 mM [Ca^2+^] at 200 Hz and in 1.5 mM [Ca^2+^] and 1 mM TEA at 100 Hz (Fig. 4g; these conditions were chosen to maximize both s_8_ and asynchronous release). By contrast, no correlation was found between s_8_ and asynchronous release measured in a later window (35–65 ms after the AP: Fig. 4g). In simulations, a negative covariance was likewise found in both conditions for the 5‒35 ms window and not for the 35‒65 ms window; furthermore, both in 3 mM [Ca^2+^]/200 Hz and in 1.5 mM [Ca^2+^]/1 mM TEA/100 Hz conditions, simulated and measured covariance values were within a factor of 2 of each other for the 5‒35 ms window (Fig. 4g). These results indicate that phasic and asynchronous release are not independent. They indicate instead that phasic and asynchronous release share the same DSs.

### Respective contributions of various SV pools during trains

**Fig. 5:**
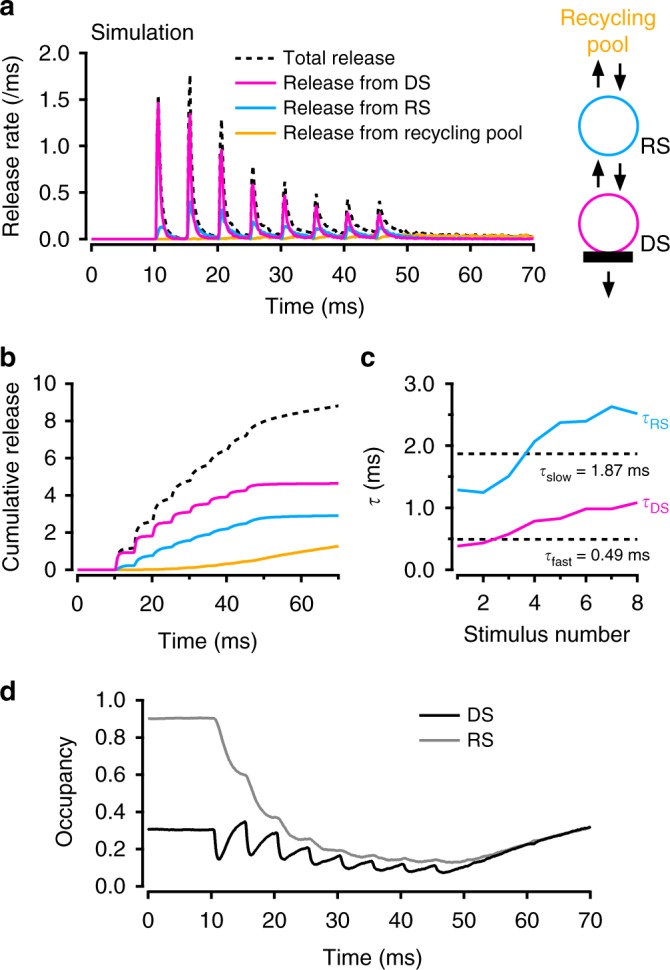
Three modes of SV release during an AP train. Monte Carlo simulations were used to trace back each released SV to its status immediately before the last AP preceding release. The simulations used a modified 2-step model incorporating inhibition of release linked to previous synapse history (Supplementary Fig. 6). **a** Having sorted out each release SV according to its status just before the AP preceding release, the total histogram of release latencies (dashed line) was decomposed into release from docking site (red), release from replacement site (blue; note slower time course of this component) and release from recycling pool (yellow). **b** Cumulated plots from **a**. **c** Monoexponential fits of the red and blue components in **b** yield time constants *τ*_DS_ (red) and *τ*_RS_ (blue). Dotted lines indicate the values of *τ*_fast_ and *τ*_slow_ for comparison. This suggests that *τ*_fast_ and *τ*_slow_ correspond to the release of SVs coming, respectively, from docking sites and from replacement sites. In addition, the two-fold increase both in *τ*_DS_ and *τ*_RS_ indicates an increasing participation of slowed release due to previous release activity. **d** Occupancy of replacement site (*ρ*) and docking site (δ) during an AP-train

The results so far indicate that release latencies differ depending on the release mode of SVs. To further investigate this correlation, we used Monte Carlo simulations to follow the fate of individual SVs in the 2-step model. Figure 5a illustrates the contributions of release events depending on three possible locations of the SV before the last release-inducing AP: docked (red), on the replacement site (blue), or in the recycling pool (yellow). Docked SVs predominate for the very first stimulations, but replacement SVs soon make a significant contribution. Their cumulated contribution reaches about 2/3 of that of docked SVs at the end of the train (Fig. 5b). Recycling SVs become significant towards the very end of the train. The shift between the 3 release modes corresponds to the 3 successive SV supply steps in the 2-step model, S_f_, R_f_ and P_r_, where S_f_ < R_f_ < P_r_. Whereas for a rested synapse, the maximum release rate for a docked SV (1.5 SVs ms^−1^ in Fig. 1f) approaches the maximum rate set by the fast P_r_ step (2 SVs ms^−1^ in the simulation of Supplementary Fig. 5a), R_f_ and S_f_ (maximum values of these rate constants are, respectively, 0.55 ms^−1^ and 0.042 ms^−1^ in Supplementary Fig. 5b) gradually restrict SV release as the AP train progresses.

Monoexponential fits to the individual increments of the plots in Fig. 5b reveal smaller time constants for docked SVs (*τ*_DS_) than for replacement SVs (*τ*_RS_). Average *τ*_DS_ and *τ*_RS_ values across an AP train are close to the *τ*_fast_ and *τ*_slow_ values determined in Fig. 3 (dotted lines in Fig. 5c), consistent with the proposal that *τ*_fast_ and *τ*_slow_ components, respectively, represent direct release from DSs and indirect release from replacement sites. In addition, both *τ*_DS_ and *τ*_RS_ increase about two-fold during an AP train (red and blue curves in Fig. 5c). The changes of *τ*_DS_ and *τ*_RS_ with stimulus number reflect a gradual slowing of the release process due to changes of P_r_, the release probability of docked SVs.

### Three separate mechanisms contribute to slowing release

In addition to the change of release mode, simulations of Figs. 3 and 4 incorporated lengthening of [Ca^2+^] decay and a change to slower release to simulate synaptic fatigue, as mentioned before (Supplementary Figs. 7 and 8). If no P_r_ change was allowed (keeping the same P_r_ profile as in Fig. 1f), the proportion of release events contributed by replacement SVs was still large (Supplementary Fig. 9a, left). However, the value of *τ*_DS_ remained constant during the AP train, and the value of *τ*_RS_ decreased during the AP train, deviating in both cases from experimental data (Supplementary Fig. 9a, right). This indicates that changes in [Ca^2+^] decay kinetics contribute to the slowing of latency distribution during trains. If changes in local [Ca^2+^] transients were included in the model but the parameters governing the release reaction remained constant, simulations became closer to experimental data but still not satisfactory as the crossover between fast and slow components did not occur in 3 mM external [Ca^2+^], 200 Hz conditions (Supplementary Fig. 9b). Thus fatigue-induced changes of P_r_ are necessary to account for the data. Altogether, it appears that 2-step release, changes in local [Ca^2+^] profile and fatigue-related slowing of release for docked SVs are all needed to explain the overall slowing process.

To clarify the functional changes of DSs during a train, time plots of the filling state of replacement site (*ρ*) and DS (δ) are illustrated (Fig. 5d). In general, changes of δ are faster than those of *ρ*, because the rate constants governing replacement–docking exchange are quicker than those governing recycling–replacement exchange. At the beginning of the train, δ drops sharply after each AP due to the loss of SVs by exocytosis, but δ quickly recovers in the following few ms due to the recruitment of SVs from the replacement site, leading to a transient overshoot over the resting value of 0.3 just before stimulation number 2. Thereafter, however, the recovery phases become gradually weaker as *ρ* decreases, so that both δ and *ρ* are drifting down together near the end of the train, before starting a slow common recovery after the end of the train.

Comparison between experimental data and simulation (Figs. 3 and 4) shows that the modified 2-step model accounts for released SV numbers and for latency distributions. As shown in Supplementary Fig. 10, it also accounts for variance-mean results, both for asynchronous release for each stimulation and for cumulative release. Altogether, the model provides a comprehensive representation of simple PF-MLI synapses under a variety of experimental conditions.

## Discussion

Using deconvolution analysis in small individual synapses, we show that the latency distribution of release events after an AP changes over a wide range and in a systematic manner depending on the previous activity of the synapse and on the amount of Ca^2+^ entry per AP. Detailed analysis suggests that latencies can be classified into three categories: fast release events that are distributed along an exponential with a time constant *τ*_fast_ near 0.5 ms; slow release events that are distributed along another exponential with a time constant *τ*_slow_ near 2 ms; and asynchronous release with latencies of 5 ms and more. Whereas for a single stimulus, only fast latencies are observed, slow and asynchronous latencies appear after the second stimulus and become more prominent as a function of stimulus number during an AP train. Increasing stimulation frequency or release probability both tend to increase the proportion of *τ*_slow_ and of asynchronous release, whereas that of *τ*_fast_ decreases.

Two mechanisms potentially accounting for a gradual lengthening of release latencies during a train are unlikely to play a significant role here. A first possible mechanism, lengthening of the AP waveform, is unlikely to play a significant role at PF-MLI synapses in view of data indicating a stable waveform^46^. A second mechanism, increasing Ca^2+^ entry, also conflicts with experimental data^22,40,46^ (Supplementary Fig. 3).

The finding of widely different values for *τ*_fast_ and *τ*_slow_ suggests two different modes of SV release. The two modes could be envisaged as reflecting two different populations of DSs, with different distances to nearest VGCC clusters (review: ref. ^4^). However, our simulations indicate that if the Ca^2+^ sensitivity of release is kept constant, the distances needed to explain the observed *τ*_slow_ values are >150 nm (Supplementary Fig. 11). Such distances are unrealistic since the arrangement of PF-MLI synapses is so compact that the proportion of free AZ membrane farther than 50 nm of nearest VGCCs is negligible (Fig. 1c; Supplementary Fig. 2). Docking and release of SVs outside the AZ is also unlikely, since SVs need to bind to AZ proteins such as RIM to be released^35^. Altogether, participation of distantly docked SVs to release is unlikely.

Another option would be that the two modes represent two kinds of SVs (or DSs) that would differ in their release probability, as proposed for primed vs. superprimed SVs^51–53^. In support for this hypothesis, superprimed SVs were found to respond faster than primed SVs to a sustained presynaptic voltage pulse^52^. However, another study did not reveal latency differences linked to superpriming, as assessed from the percentage of asynchronous vs. synchronous release^51^. Overall, the links between superpriming and release latencies remain to be investigated.

The appearance of a second component during AP trains suggests two kinetic processes in series. We propose that, whereas fast release events represent the classically envisaged release of docked SVs, slow release events represent sequential docking-fusion events (2-step release) involving SVs that were bound to the replacement site prior to the response-triggering AP. In this view, the difference between *τ*_fast_ and *τ*_slow_ reflects the extra time that is taken by the SV to first move to the DS before being released. For isolated stimuli under standard conditions, the movement of replacement SVs towards the DS occurs too late to overlap significantly with the AP-induced [Ca^2+^] transient, so that only a *τ*_fast_ component is observed. But if R_f_ increases due to strong or repeated Ca^2+^ entry and/or if the [Ca^2+^] transient is prolonged by combination with earlier transients, newly docked SVs may find a large enough tail of [Ca^2+^] to undergo exocytosis, giving rise to the *τ*_slow_ component.

Several experimental findings are in line with the 2-step docking/fusion proposal for the *τ*_slow_ component. The dependence of the *τ*_fast_ to *τ*_slow_ crossover point on stimulation frequency (Fig. 4e, f) is in accord with the expectation that the mean global [Ca^2+^], and hence R_f_, increases with stimulation frequency, since the amplitude of the *τ*_slow_ component follows the increase of R_f_. The sensitivity of the crossover point to latrunculin (Fig. 3e) is in agreement with the sensitivity of R_f_ to latrunculin^22^. Finally, as expected, increasing Ca^2+^ entry (either by increasing the external [Ca^2+^] or by applying TEA) enhances both δ decrease and R_f_ increase, resulting in an acceleration of the crossover (Fig. 4e, f).

The critical need of an overlap between docking and [Ca^2+^] decay offers an explanation for the fact that the *τ*_slow_ component of release has not been reported before. In the calyx of Held, AP-induced release is largely carried by the so-called fast-releasing pool (FRP) of vesicles^54^. The FRP can be subdivided into a ‘superprimed’ pool and a normally primed one^53^, the former releasing during an AP with high probability (≈0.6) and being replaced quite slowly. If, as suggested here for PF-MLI synapses, the progression from normally primed to superprimed is seen as a maturation process, the question arises as to why release latencies change only very little during AP trains in the calyx of Held. A likely explanation may reside in the relationship between AP-induced [Ca^2+^] transients and replacement kinetics. Later in the train, release will be dominated in the calyx by vesicles that reach the mature state not during [Ca^2+^] transients but in the inter-AP interval. Their latencies will reflect the time course of [Ca^2+^] transients. By contrast in PF-MLI synapses, the *τ*_slow_ component may be seen as direct recruitment and subsequent release during [Ca^2+^] transients. Thus the higher prominence of *τ*_slow_ release in PF-MLI synapses compared to the calyx of Held could simply reflect faster replacement kinetics (R_f_) in the former preparation. This difference between PF-MLI and calyx of Held synapses may reflect different adaptations to distinct functional constraints. The entire sensory pathway driven by cerebellar mossy fibres, including mossy fibre-granule cell and PF-MLI synapses, handles relatively short AP bursts at high frequency^12,55^. As argued in previous studies^13,14,22,56^, maintenance of synaptic strength during these bursts relies on very fast SV replenishment. The present study suggests that this maintenance of transmission occurs at the expense of some loss of time precision. By contrast, the hearing sensory pathway including the calyx of Held synapse relies on precisely timed signals during prolonged, high frequency trains, and in this case, time precision is more important than global synaptic strength.

According to our simulations, the facilitation observed in paired stimulations results from a combination of an increase in P_r_ following local [Ca^2+^] accumulation, from an increase in the *τ*_slow_ component and from an increase of δ following fast SV recruitment^22^. This facilitation decays as a function of interpulse interval within 80 ms (Fig. 2a), raising the question of how δ returns to its baseline value. As shown in Fig. 2d, the extent of asynchronous release observed between the two stimulations is insufficient to account for the decay of δ. This leads us to suggest that the decay of δ is linked to a reversion of the binding step R_f_ (backward step R_b_). Docking reversibility has been proposed on the basis of FM 1-43 imaging experiments^57^ and is consistent with recent evidence indicating reversibility of some of the steps leading to exocytosis (‘de-priming’: ref. ^58^). While we assume an R_b_ step leading back from DS to replacement site, we cannot exclude that docked SVs leave the DS directly into the cytosol instead of returning to the replacement site. We prefer the option of returning to the replacement site because the returned SV can then immediately be reused for docking, whereas a SV that would drift in the cytosol would require a longer recycling route.

A re-equilibration of δ following the decay of global [Ca^2+^] accounts for the observation of a simultaneous return of PPR and covariance values to baseline as a function of interpulse interval (Fig. 2c, e), because these two parameters depend on δ. A similar explanation holds also for the simultaneous return of the amplitude of the *τ*_slow_ component, since this component largely depends on the elevation of R_f_ linked with the global [Ca^2+^] (Supplementary Figs. 4 and 12). A recent electron microscopy study using flash-and-freeze technique revealed that in certain mutants of synaptotagmin 1 (with modified C2B region), the number of SVs located 0–5 nm away from presynaptic AZs transiently increased 10 ms after a presynaptic AP and decayed back with a time course similar to that of paired-pulse facilitation^23^. Considering the similar reversibility of our δ change and its relationship with paired-pulse facilitation, these results suggest that a δ change in the 2-step model might reflect a morphologically observable change in the number of docked SVs.

Given the very fast kinetics of 2-step release, where docking and release occur within ∼2 ms, it seems likely that SVs are already engaged with the release apparatus when they are located at the replacement site. Thus our results, together with those of Chang et al.^23^, suggest that docking and release are not strictly separated processes and that they are much more integrated together than hitherto envisaged. Altogether, the distinction between docked and replacement SVs in both morphological and molecular terms must await future studies.

Asynchronous release grows more slowly than slow release, and it is mainly observed after the end of the train. In the overall response to 8 AP trains at 200 Hz with 3 mM external [Ca^2+^], fast and slow release make comparable contributions to the total synaptic output, while asynchronous release makes a smaller but significant contribution (Fig. 3b, e).

Even though fast, slow and asynchronous release are readily distinguishable, our results and simulations indicate that these three modes of release are closely related. In particular, our latrunculin results (Fig. 3b) suggest that phasic release and asynchronous release both depend on cytoskeleton-driven SV docking; our covariance results (Fig. 4g) show that phasic release and asynchronous release share the same pathway; and our simulations indicate that there is no need to assume different sensitivities to [Ca^2+^] for phasic release and for asynchronous release. These results suggest that both phasic and asynchronous release result from the flow of identical SVs through a single release machinery. For the first stimulation, release kinetics mainly reflects the ultimate exocytosis step, but as the AP train progresses, release kinetics increasingly involves slower, more upstream steps, namely docking (slow release) and supply from the reserve pool (asynchronous release). The transition from fast to slow release, as well as that from slow release to asynchronous release, appear as successive adaptations of the synapse to maintain its output during prolonged stimulations, at the expense of a loss of time accuracy following DS depletion and AZ cluttering. At each transition, some degree of time accuracy is abandoned for the sake of maintaining synaptic responsiveness.

The present proposal that a single release apparatus is responsible for several aspects of synaptic function including initial fast release, facilitation-associated slowing of release and asynchronous release may help to interpret the functional role of certain synaptic proteins. In particular, the initial suggestion that genetic ablation of synaptotagmin 7 in central synapses selectively inhibits SV replenishment^59^ has been challenged by subsequent reports that it rather inhibits synaptic facilitation^60^ or asynchronous release^61^, leading to the speculation that synaptotagmin 7 may target different processes in different synapses. Very recent studies suggest, however, that synaptotagmin 7 exerts a mixed action on replenishment, facilitation and asynchronous release in the same synapses^62^, and such a mixed action occurs at PF-MLI synapses^63^. These intriguing results may be explained within the framework of the 2-step release model if the primary synaptotagmin 7 target is the Ca^2+^-sensitive transition between replenishment site and DS^59^ (R_f_). In such a case, synaptotagmin 7 ablation would affect synaptic facilitation and asynchronous release in addition to SV replenishment, since the R_f_ step participates in all three processes.

## Methods

### Recording procedures

Sagittal slices (200 μm thick) were prepared from the cerebellar vermis of Sprague-Dawley rats (P12‒P16 and P26‒P29) following the animal care guidelines of Paris Descartes University (approval no. A-750607). Recordings were from MLIs, comprising about 2/3 of basket cells and 1/3 of stellate cells. The composition of the extracellular solution was (in mM): 130 NaCl, 2.5 KCl, 26 NaHCO_3_, 1.3 NaH_2_PO_4_, 10 glucose, 2 CaCl_2_, and 1 MgCl_2_ (osmolarity: 300 mosm). This solution was equilibrated with 95% O_2_ and 5% CO_2_ (pH 7.4). The internal recording solution contained (in mM): 144 K-gluconate, 6 KCl, 4.6 MgCl_2_, 1 EGTA, 0.1 CaCl_2_, 10 HEPES, 4 ATP-Na, 0.4 GTA-Na; pH 7.3 (osmolarity: 300 mosm). Recordings were at 32‒34 °C.

### Simple synapse recording

Recordings of simple PF-MLI synapses were obtained under voltage clamp at −60 mV. NMDA receptors and GABA_A_ receptors were blocked by inclusion of D(‒)-2-amino-5-phosphonopentanoic acid (50 μM) and gabazine (15 μM). Procedures to find an appropriate location for electrical stimulation were as described^38^. Briefly, we puff-applied the internal solution including 150 mM K^+^ from a pipette using small pressure steps while moving the pipette in the granule cell layer. When we found a burst-like EPSC response in the postsynaptic cell, we reduced the pressure of puffing to better define the spot for stimulation. Then we switched to electrical stimulation, using the same pipette, and we adjusted stimulation intensity to fire a connected granule cell under minimal stimulation conditions. Definitive acceptance of the experiment as a usable simple synapse recording occurred after analysis and depended on three criteria^31^: (i) a decrement of EPSC amplitudes of second events in a pair, reflecting activation of a common set of receptors belonging to one postsynaptic density; (ii) a Gaussian distribution of EPSC amplitudes with a coefficient of variation (CV) <0.5; and (iii) stability of the overall responsiveness over time. Specifically, in our experiments amplitude occlusion of first EPSC pairs (*ω*) ranged from 0.264 to 1 (median = 0.648). The time constant of recovery from the occlusion ranged from 0.266 to 7.22 ms (median = 1.50 ms). CV for the distribution of EPSC amplitudes ranged from 0.22 to 0.39 (mean ± sd = 0.307 ± 0.050). A slope of plots of the number of events in a train as a function of sweep number ranged from −0.137 to 0.099 events per train (mean ± sd = −0.026 ± 0.064). Single stimulations and trains of two or eight stimulation pulses were applied repetitively with intervals of 10 s between sweeps. Statistical data were derived from sequences of 10‒30 trains.

### Decomposition of EPSCs

We determined occurrence times of individual EPSCs based on deconvolution analysis, as detailed in Malagon et al.^31^, and we built latency distributions by averaging the occurrence times across experiments. We briefly describe the analysis here. First, we made an average of single EPSCs obtained during asynchronous release to obtain a template in a given synapse. Then the average miniature EPSC (mEPSC) was fit by triple-exponential function with five free parameters (rise time, amplitude, fast decay time constant, slow decay time constant and amplitude fraction of slow decay). Next, mEPSC and individual data traces were deconvolved using the five parameters. The deconvolved mEPSC resulted in a narrow spike, and the deconvolved data traces resulted in sequences of spikes. Finally, we fit a given deconvolved trace by a sum of scaled narrow spikes in order to obtain the timing of each event. The amplitude parameter was free because the peak EPSC amplitude varied during a train due to receptor saturation and desensitization. The above procedure had a detection limit that caused a failure of separation of two events occurring within 0.2 ms. To correct for missed events, we split into two the events having amplitude 1.7 times larger than the average amplitude obtained during asynchronous release.

### Pharmacological manipulations

In LatB experiments, data were collected at least 5 min after switching the bath solution to the test solution. In TEA experiments, we checked somatic potassium currents by applying 3-ms voltage step to 0 mV until TEA reduced potassium current amplitudes to a stable level, and then we started to collect data.

### Ca^2+^ imaging of presynaptic varicosities

For Ca^2+^ imaging experiments, sagittal (200 μm) slices were prepared using a modified extracellular saline, as detailed in Brenowitz and Regehr^42^. Experiments were conducted in the same conditions as in the electrophysiology experiments (32‒34 °C, with the 3 mM extracellular Ca^2+^ saline including APV and gabazine). Granule cells were loaded under whole-cell recording with a solution containing (in mM): 140 K-gluconate, 5.4 KCl, 4.1 MgCl_2_, 9.9 HEPES, 0.36 Na-GTP, 3.6 Na-ATP, 500 μM of Oregon green 488 BAPTA-6F (OGB-6F: *K*_d_ for calcium of 5.1 μM; Invitrogen), and 20 μM Alexa-594 (Invitrogen). Imaging was performed with a custom-built 2-photon system with 820 nm excitation provided by a MaiTai-Sapphire laser (Spectra Physics, USA). To visualize the granule cell axon, large raster scans were performed while acquiring the Alexa 594 fluorescence with a red channel photomultiplier (Hamamatsu H7422 PA-sel, bandpass emission filter 635 ± 65 nm, Chroma Technology; or avalanche photodiode Perkin Elmer, SPCM-AQR-13). Single varicosity imaging was performed using raster scans of 5 by 2 μm dimensions at dwell times of 2 ms. The granule cells were kept under current clamp conditions. We found that keeping a hyperpolarized holding potential improved recording stability, so that resting membrane potential was kept around ‒90 mV. APs were evoked by 1-ms steps of 350‒500 pA. Stimulation protocols were 4 or 8 APs at 100 Hz and were repeated every 1 min. Calcium signalling was analysed in the pixels encompassing the varicosity in terms of fluorescence changes relative to prestimulus values (ΔF/F_o_, expressed in %) with software written in the IGOR-Pro programming environment (Wavemetric, Lake Oswego, OR, USA).

The calibration procedure used to convert ΔF/F_o_ values into intracellular [Ca^2+^] is explained in detail in Miki et al.^22^. This procedure gave a slope of 0.32 μM per AP in 3 mM extracellular [Ca^2+^] (Supplementary Fig. 3). This slope was then corrected as before^22^ to compensated for the buffering capacity of OGB-6F, resulting in 0.89 μM per AP. This value is similar to the peak global [Ca^2+^] obtained in simulations (Fig. 1d, right).

### Latency distribution analysis

We obtained the latency distribution of release events based on deconvolution analysis. We then separated this distribution into three components: fast synchronous release, slow synchronous release, and asynchronous release. To separate asynchronous release, we first deleted release events occurring during the first 4.6 or 5 ms following each AP for 200 Hz or 100 Hz experiments, respectively. This left only late events occurring 4.6–5 ms after each AP (at 200 Hz) or 5–10 ms after each AP (at 100 Hz). These remaining data were filtered, and gaps were filled in using connecting straight lines (black in panels for asynchronous release in Supplementary Fig. 5 and Supplementary Fig. 6a). After further smoothing, we obtained the release rate for asynchronous release (red in Supplementary Fig. 5 and Supplementary Fig. 6a). We calculated the release rate for synchronous release by subtracting asynchronous release from the initial distribution (‘subtracted’ panels in Supplementary Fig. 5 and Supplementary Fig. 6a). Finally, we separated fast and slow components of synchronous release by double exponential fits of cumulative latency distributions for each stimulation. Time constants are given together with 95% confidence intervals as obtained from IGOR-Pro routines.

When performing latency analysis of group experiments, the results of individual cells were slightly time shifted, if needed, to compensate for timing differences in presynaptic APs across experiments. Such readjustments never exceeded 1.2 ms.

### Covariance analysis

For the analysis of Fig. 2e, covariance was calculated as: <s_1_ - <s_1_ >><s_2_ - <s_2_>>, where s_1_ and s_2_ are SV counts obtained in 5 ms long time windows following AP number 1 and 2, respectively. Exceptionally, in the case of 3 ms interval, we used 3 ms long time windows following AP 1 and 2.

For the analysis of Fig. 4g, SV counts were taken on one hand as the sum of SV release numbers in a 5 ms long time window following AP number 8, and on the other hand of the sum of SV release numbers in a 30 ms long time windows following AP number 8 (either from 5 to 35 ms following that AP or from 35 to 65 ms following that AP).

### Simulation

*Simulations of Ca*^*2+*^ *buffered diffusion*: To calculate the spatiotemporal distribution of [Ca^2+^] in the vicinity of VGCC clusters at a PF bouton, we used a Java-based 3D diffusion-reaction simulator D3D running on a Windows 7 operating system^19^. Ca^2+^ entry, diffusion and buffering were simulated by numerically integrating differential equations using an explicit finite-difference (Euler) method with a fixed time step (0.03 μs) and an elementary integration volume (i.e. voxels). Simulation voxel was a 10 × 10 × 10 nm^3^ cube. The total simulation volume was a 0.9 (*x*) × 0.5 (*y*) × 0.5 (*z*) μm^3^ cuboid, matching the size of the bouton volume^64^. Simulation voxel was a 10 × 10 × 10 nm^3^ cube. The four surfaces were set to reflect all diffusants. A single Ca^2+^ entry site was placed in the centre of a single surface (*z* = 0) bounding this volume. The Ca^2+^ entry site was composed of three channel clusters, each having nine channels. The nearest neighbour distance between channel clusters (centre to centre) was 126 nm, and the nearest neighbour distance between channels within a cluster was 20 nm (Fig. 1c). This ‘synthetic’ channel distribution is based on observed distributions of immunogold-labelled Cav2.1 in the PF boutons onto MLIs observed using sodium dodecyl sulphate-digested freeze fracture replica labelling^38^. We also simulated local [Ca^2+^] change using a real immunogold distribution directly taken from the replica labelling (Supplementary Fig. 1a), finding a similar Ca^2+^ waveform (Supplementary Fig. 2c, d). In control condition (3 mM [Ca^2+^]), the amplitude of Ca^2+^ influx through each channel during an AP was set at 0.2 pA, considering a single channel current of Cav2.2 at −65 mV of 0.33 pA (ref. ^65^) and a fraction of channel open probability during an AP of ~0.7 (ref. ^66^). We assumed a Gaussian-shaped Ca^2+^ influx with a half-amplitude duration of 0.34 ms (ref. ^67^). Other model parameters were obtained from previous recordings from PF boutons if possible or else from other presynaptic terminals as follows: Ca^2+^ diffusion coefficient *D*_Ca_ = 220 μm^2^ s^−1^ (ref. ^68^), resting free [Ca^2+^] = 50 nM, endogenous buffer concentration = 2 mM, endogenous buffer *K*_D_ = 50 μM, and endogenous buffer forward binding rate constant K_on_ = 2 × 10^8^ M^−1^ s^−1^ (ref. ^67^). We also included 200 μM free ATP in the simulation^19^. The values for Ca^2+^ binding to ATP were: *K*_D, Ca_ = 200 μM, K_on, Ca_ = 5 × 10^8^ M^−1^ s^−1^ (ref. ^69^), *D*_ATP_ = 220 μm^2^ s^−1^. Ca^2+^ extrusion via active transport was included on all surfaces at a rate of 0.9 Ca^2+^ ms^−1^ (ref. ^70^). To match the decay of measured (Ca^2+^ imaging) and simulated global [Ca^2+^], we adjusted the concentration of calretinin, which is a major endogenous buffer in PF boutons^39^. Although 30 μM best reproduced Ca^2+^ recovery data, we adopted a calretinin concentration of 100 μM, as release rates were almost identical within 100 ms after AP train onset for 30 and 100 μM calretinin. In these simulations, the additional buffering capacity associated with the presynaptic pipette solution (OGB-6F) were taken into account. Kinetics of Ca^2+^ binding to calretinin is from Fass et al.^71^. Simulation parameters for standard conditions (unmodified presynaptic solution) are summarized in Supplementary Table 1.

*Simulation of 2-step model*: We calculated the release rate by numerical integration of differential equations for single and paired-pulse experiments using Igor Pro with a time interval of 0.01 ms (Figs. 1 and 2). Monte Carlo simulations of SV release were performed for single, paired and eight pulse experiments using Igor Pro with an interval of 0.01 ms (Fig. 3‒5, Supplementary Figs. 9, 11 and 12). In all simulations, we set the number of DSs to 4. In Fig. 1, we used a simple model without DS replenishment as shown in Fig. 1e. This simple model has two parameters, δ and P_r_. We fixed the initial value of δ at 0.3. As in our previous report^22^, we obtained δ by minimizing the sum of least square deviations of variance-mean results during trains. The discrepancy between the value of 0.3 and our previous estimate of 0.45 arises, because in our present model, contrary to the previous simplified version of the model^22^, newly recruited vesicles between stimulations can release before the next stimulation. This new feature accounts for the difference in δ values. To calculate P_r_, we fitted experimental data using the allosteric model of Lou et al.^41^, providing the parameter values: K_on_ = 5 × 10^8^ M^−1^ s^−1^, K_off_ = 5000 s^−1^, b = 0.75, γ = 2100 s^−1^, and f = 31.3 (Fig. 1f). γ is the fusion rate of the 5Ca-binding state (V_5Ca_), which is identical to l_+_ × f^5^. f is a factor determining the increase in vesicle fusion rate upon Ca^2+^ binding. The fusion rate at V_0Ca−4Ca_ is given l_+_ × f^0‒4^. b is a cooperativity factor^41^. This procedure was based on the local [Ca^2+^] obtained from Ca^2+^ simulation at 40-nm distance from the nearest Ca^2+^ channels.

In Fig. 2 (paired-pulse experiments), first we tried to keep the values of the parameters for the allosteric model for P_r_. Nevertheless, as the average release probability values for the first AP were slightly different for the experiments of Fig. 2 from those obtained in the experiments of Fig. 1, the value of the parameter γ was readjusted. Second, we added a replenishment step R_f_ to the simple model following a Michaelis–Menten reaction having two free parameters, V_max_ and *K*_d_, as shown in Fig. 2b and Supplementary Fig. 4. Fitting the release rate of paired-pulse experiments by changing parameters of Michaelis–Menten reaction provided a V_max_ value of 800 s^−1^ and a *K*_d_ of 2 μM for the forward reaction rate (R_f_). The backward reaction rate (R_b_) was set to keep a δ value of 0.3 at a resting [Ca^2+^] of 50 nM.

In the simulations for eight pulse experiments (Figs. 3‒5), we used the full model with recruitment from recycling pool to replacement site (Fig. 3d). According to our previous model^22^, the possibility was kept open that SVs would occupy both one DS and its associated replacement site simultaneously. As for R_f_ (see above), we defined the recruitment step S_f_ using a Michaelis–Menten reaction having two free parameters, V_max_ and *K*_d_, that were determined in order to fit experimental data while keeping the parameter values for P_r_, R_f_ and R_b_ (Supplementary Fig. 7; V_max_ = 60 s^−1^, *K*_d_ = 2 μM for S_f_). The backward reaction rate (S_b_) was set to keep a δ value of 0.3 and a δ value of 0.9 at a resting [Ca^2+^] of 50 nM. To improve fitting results for late responses in trains, we introduced a slow P_r_ having 10 times reduced K_on_ and 2.5 times reduced K_off_ compared to normal P_r_ (Supplementary Figs. 7 and 9). The first two release events in one AZ used the high P_r_ parameters. The next events used the high P_r_ release parameters if they happened in a DS that had not released yet and the slow P_r_ release parameters in the other case. We set a recovery time of 40 ms for each DS during which the parameters were linearly extrapolated between slow P_r_ and normal P_r_. Therefore, if there is no release for 40 ms after previous release in the DS, P_r_ recovers its normal parameter values. In the simulation for LatB experiments, we set the recovery time to 1 s, in accord with our previous findings that LatB increases the duration of the refractory period^22^. In the simulation of 1.5 mM [Ca^2+^] experiments, we used a δ value of 0.15 instead of 0.3 at rest (Supplementary Fig. 5), in accord with unpublished results showing that the resting δ value varies roughly proportionally with external [Ca^2+^] in the 1.5–3 mM concentration range (Malagon et al., in preparation).

In Monte Carlo simulation, P_r_, R_f_, R_b_, S_f_ and S_b_ were first calculated from simulated [Ca^2+^] and from the initial occupancy of DS and replacement site as above. The amount of release and occupancies in 4 DSs were obtained with a time interval of 0.01 ms. We performed the calculation 5000 times in a given condition and used the averaged value as result.

### Code availability

All relevant codes are available from the authors.

## Electronic supplementary material

Supplementary Information

Peer Review File

## Data Availability

All relevant data are available from the authors.
