# Supplementary material for: Two-component latency distributions indicate two-step vesicular release at simple glutamatergic synapses
Source: Nat Commun. 2018 Sep 26;9:3943. doi: 10.1038/s41467-018-06336-5 (PMC6158186; doi:10.1038/s41467-018-06336-5)
Supplement: Supplementary file 1 — Supplementary Information [file 41467_2018_6336_MOESM1_ESM.pdf]

Supplementary Information

Two-component latency distributions indicate two-step vesicular release at simple glutamatergic synapses

Miki et al.

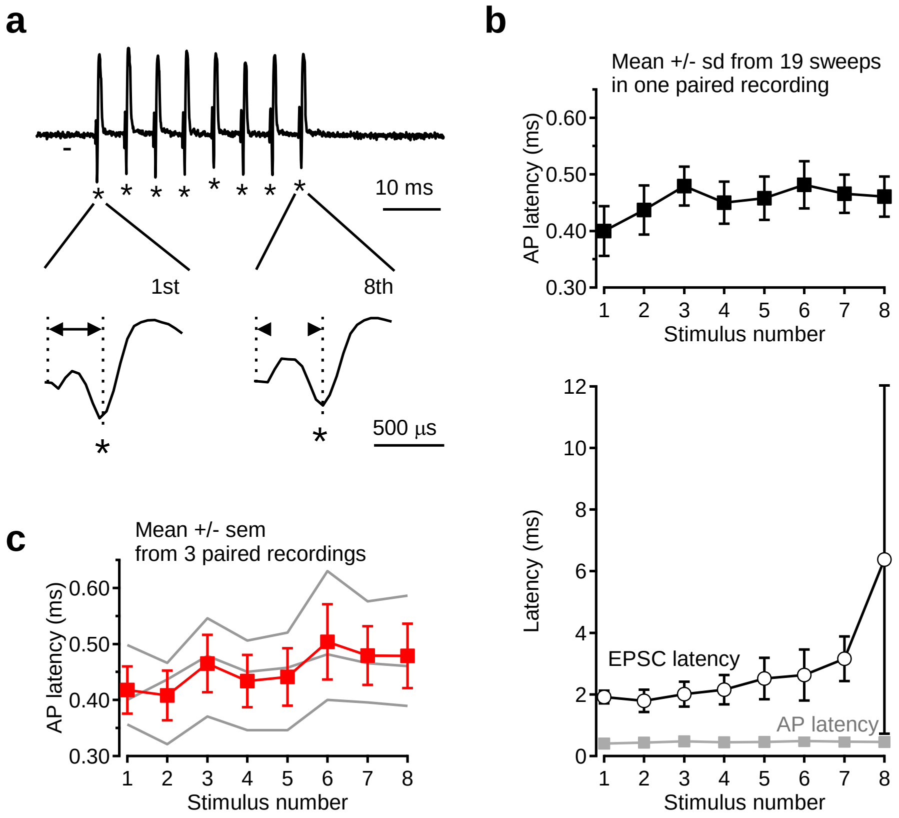

Supplementary Figure 1: Latencies of APs and release events in paired recordings
Latencies of APs and release events were detected by paired recording. Whole cell recording and cell-attached recording were performed in connected postsynaptic MLI and presynaptic GC soma.

a: A representative trace of cell-attached recording from a GC soma. 1 ms voltage steps were applied at 200 Hz. Peaks of AP-associated currents are shown by asterisks. These peaks correspond to the time point of maximum slope of the presynaptic AP[1]. We measured the time difference between the onset of voltage step and the peak of AP-associated currents (arrows in bottom panels).

b: Top, plot of AP latency (mean ± sd) vs. stimulus number, showing little jitter at a given stimulus number, and little variation of the mean latency during an AP train. Bottom, in contrast to AP latencies (grey squares, same as top panel), EPSC latencies (open symbols; mean ± sd) display significant jitter at each stimulus number, and their mean value increases with stimulus number. This plot displays the latencies of first events after each stimulation; an all-latency plot would display even broader scatter.

c: Group analysis of AP latencies from 3 paired recordings (mean ± sem). Gray lines indicate averages of individual paired recordings.

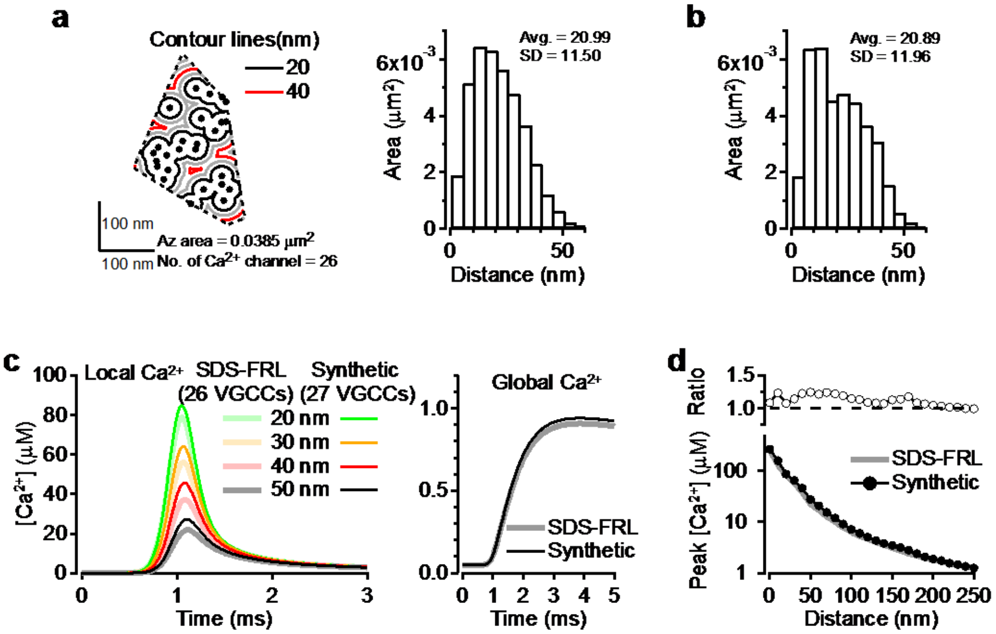

Supplementary Figure 2: Ca^{2+} channel distributions in AZ

a: Left: A representative example of Ca^{2+} channel labeling in a single PF-MLI AZ of a SDS-treated freeze fracture replica (SDS-FRL). Same data pool as in Miki et al.[2]. We chose an AZ that displayed an area close to the average (0.0427 μm^2), and a number of Ca^{2+} channel close to the average number corrected by labeling efficiency[2] (27). Contour lines show distances from nearest Ca^{2+} channel. Dashed line shows the borders of the AZ area. Right: Histogram of area distribution of this representative data as a function of distance from nearest Ca^{2+} channel in the AZ region.

b: Histogram of area distribution of the model AZ in Figure 1c. Note the similarity with the histogram in **a**. We used the slightly idealised version of Figure 1c in our simulations, because the 3-fold rotation symmetry of the distribution in Figure 1C facilitated the calculations of Ca^{2+} diffusion.

c: Comparison of $[Ca^{2+}]$ waveforms calculated using the VGCC distribution in A and the model VGCC distribution of Fig. 1c.

d: Comparison of peak amplitudes of $[Ca^{2+}]$ waveforms between VGCC distribution from SDS-FRL data and model VGCC distribution. Upper: Ratio of peak $[Ca^{2+}]$ from model VGCC distribution to that from the SDS-FRL representative data. Lower: Peak $[Ca^{2+}]$ as a function of distance from nearest VGCC, for the two distributions.

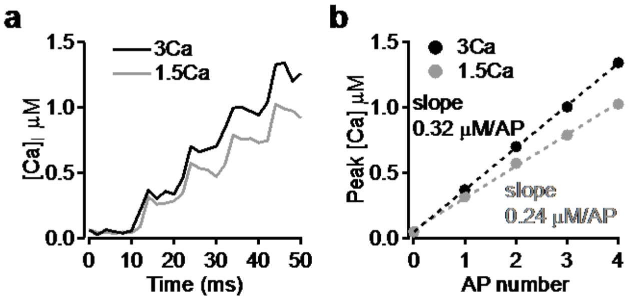

<u>Supplementary Figure 3</u><u>: 2-photon Ca^{2+} imaging in PF varicosities</u>
a: Time course of presynaptic intracellular [Ca^{2+}] in 1.5 and 3 mM extracellular [Ca^{2+}] conditions when applying 4 APs at 100 Hz.
b: Peak [Ca^{2+}] as a function of AP number in 1.5 and 3 mM extracellular [Ca^{2+}]. Linear fits have slopes of 0.24 and 0.32 μM/AP for 1.5 and 3 mM [Ca^{2+}] respectively. The difference between the value of 0.32 μM/AP in 3 mM extracellular [Ca^{2+}] obtained here and the value of 0.90 μM/AP used for [Ca^{2+}] simulation in Fig. 1d arises from the presence of exogenous buffers associated with the recording solution in the present experiments (see Methods).

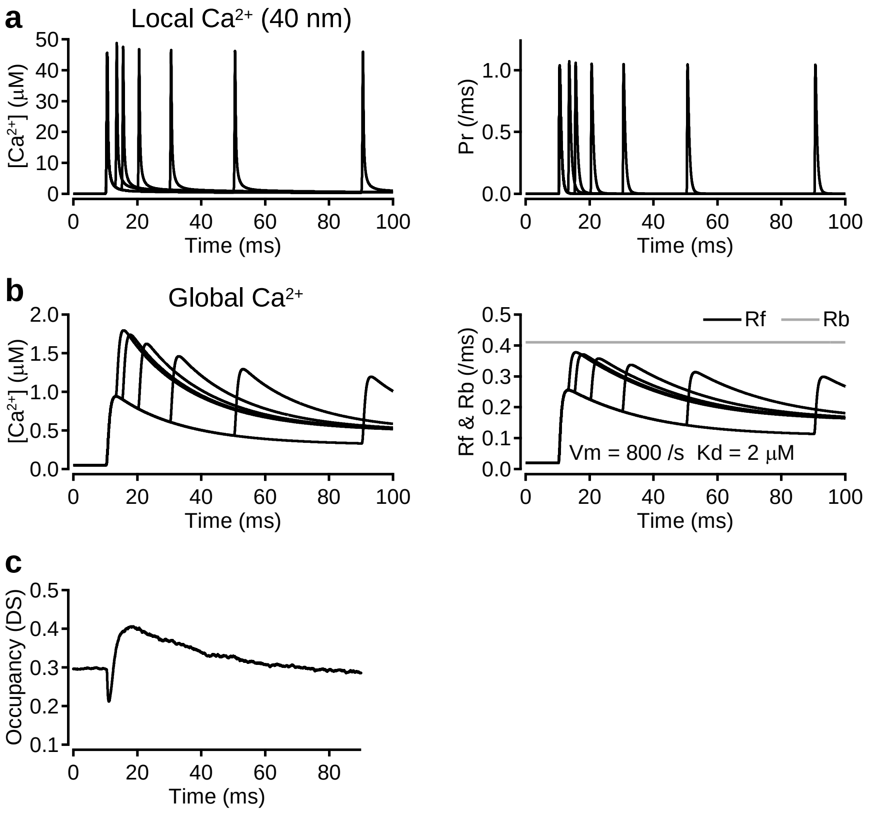

<u>**Supplementary Figure 4**: Values of parameters for the simulation of paired-pulse experiments</u>

a: Left panel: Local [Ca^{2+}] waves at 40 nm distance from Ca^{2+} channel clusters for paired pulse stimulations with different inter-AP intervals. Right panel: P_r were calculated from local [Ca^{2+}] waves using allosteric model with parameter values k_{on} = 5 x 10^8 $M^{-1}s^{-1}$, k_{off} = 5000 s^{-1}, b = 0.75, g = 1500 s^{-1}, f = 31.3.

b: Left panel: Global [Ca^{2+}] waves for paired pulse stimulation with different intervals. Global [Ca^{2+}] was defined as the average of [Ca^{2+}] in x-y plane at z of 0.25 µm in the simulation bouton with a volume of 0.9(x) x 0.5(y) x 0.5(z) µm (Fig. 1c). Right panel: Replenishment rates from replacement site to docking site (R_f: forward rate, R_b: backward rate). R_f is calculated using Michaelis-Menten kinetics based on the global [Ca^{2+}] profile shown in the left panel, assuming a maximum rate V_m = 800/s and a K_d of 2 µM. While this treatment is in conformity with previous modeling of R_f (ref. 3), another option would be to calculate R_f based on the quickly evolving local [Ca^{2+}] prevailing near the replacement site, at a distance of 45-90 nm from the plasma membrane, and to introduce a downstream rate limiting step to represent actual SV movement. We preferred the simpler first option to the second, potentially more relevant modeling because it contained a smaller number of free parameters.

c: Docking site occupancy (δ) displays a quick decrease followed by a rebound and an overshoot over the basal value within 5 ms. Then it slowly decays back to the resting state.

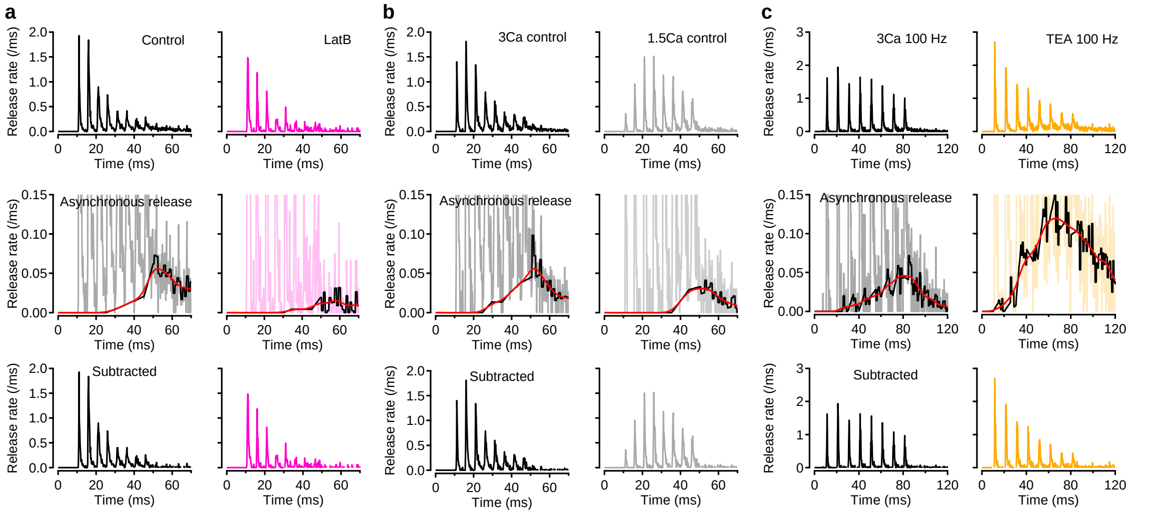

Supplementary Figure 5: Effects of various experimental manipulations on release rates during trains

Overall release rates (upper row) have been decomposed into a superslow component representing asynchronous release (middle row; expanded vertical scale) and a phasic component (lower row). To extract superslow component, we blanked release rates during 4 ms after every AP, filtered remaining points with a 5 points window (black trace in middle row) and performed smoothing (red trace in middle row).

a: Comparison between control synapses (3 mM external [Ca^{2+}] and 200 Hz stimulation) and synapses pretreated with latrunculin B.

b: Comparison between 1.5 mM and 3 mM external [Ca^{2+}] (200 Hz stimulation in each case).

c: Comparison between control recordings (3 mM external [Ca^{2+}] and 100 Hz stimulation) and test recordings obtained after adding 1 mM TEA to increase release probability.

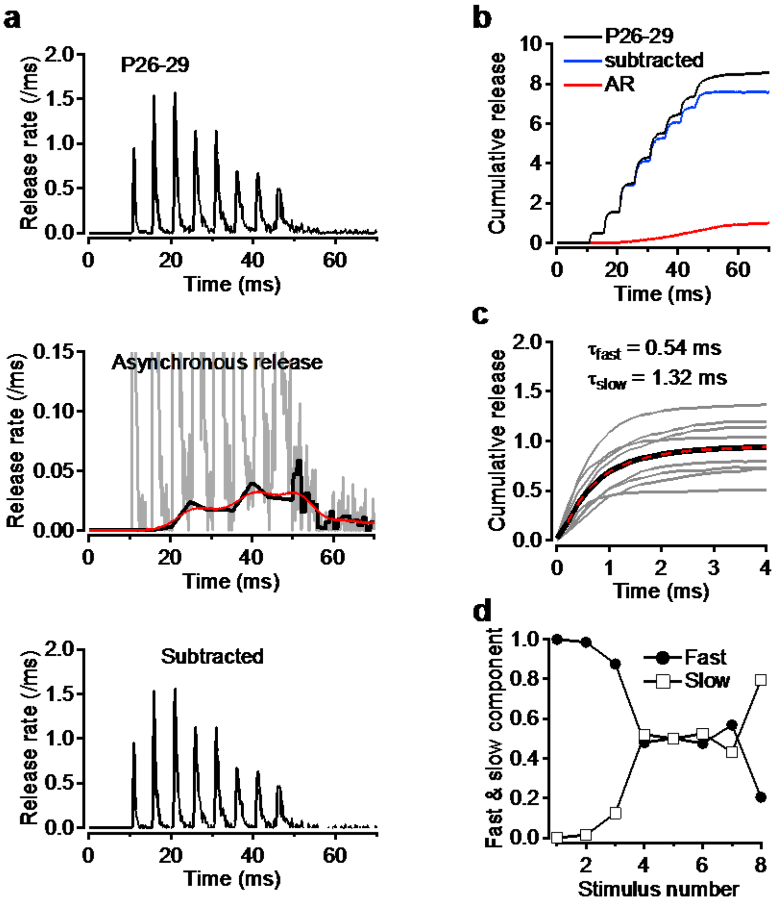

Supplementary Figure 6: Time course of fast, slow and asynchronous release during AP trains in 4 weeks old rats

a: Average release rate (per ms and per simple synapse; 3mM external [Ca^{2+}] and 200 Hz stimulation; n = 10 experiments). Middle panel: Expanded vertical scale highlighting asynchronous release. Lower panel: Average release rate after subtracting asynchronous release from original average release rate.

b: Cumulative latency counts.

c: Superimposed cumulative latency distributions for each AP stimulation during 8-AP train. Average traces of 8-AP responses (thick black curve) is fitted with a double exponential (red) with indicated τ_{fast} and τ_{slow} values.

d: Relative contributions of τ_{fast} and τ_{slow} component as a function of stimulus number.

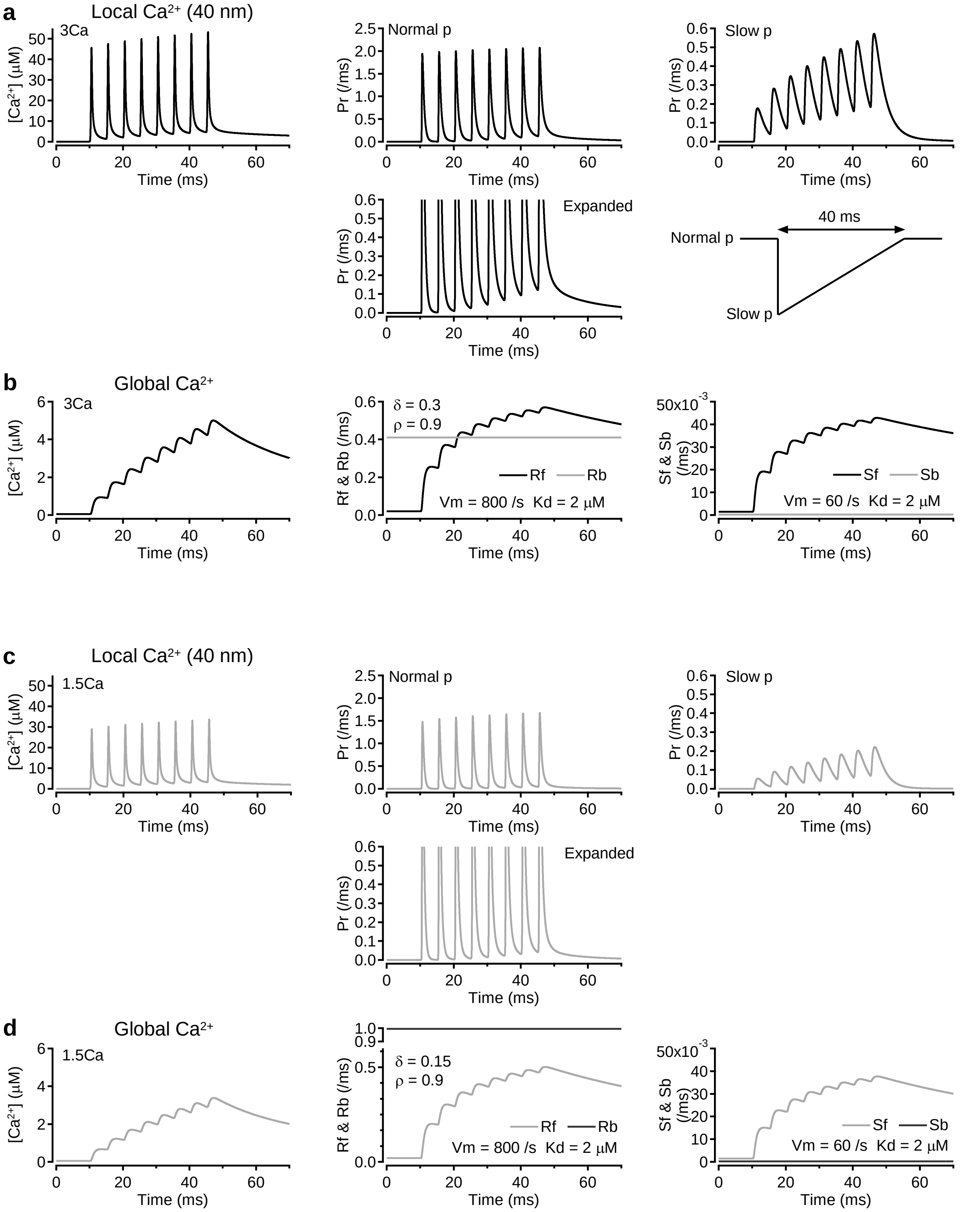

<u>**Supplementary Figure 7**: Parameter values for the simulation of 8-pulse experiments in 3 and 1.5 mM external [Ca^{2+}] conditions</u>

a: Average [Ca^{2+}]$_i$ wave at a 40-nm distance from the nearest Ca^{2+} channels for 200 Hz stimulation in 3 mM external [Ca^{2+}] condition (3Ca; left). P_r was calculated from local [Ca^{2+}]$_i$ using the standard allosteric model (k_{on} = 5 x 10^8 $M^{-1}s^{-1}$, k_{off} = 5000 s^{-1}, b = 0.75, γ = 2800 s^{-1}, f = 31.3 ; upper middle). The bottom middle panel shows a vertically expanded plot of P_r, illustrating the widening and accumulation of late

release rate as a function of stimulus number. Slow P_r was calculated starting from the local $[Ca^{2+}]_i$ profile shown in the left panel, using modified parameter values of the allosteric model (k_{on} = 5 x 10^7 $M^{-1}s^{-1}$, k_{off} = 2000 s^{-1}, b = 0. 5, γ = 2000 s^{-1}, f = 31.3 ; right). The lower right panel shows a diagram illustrating the return from high P_r release to low P_r release, where each parameter linearly changes from its high P_r value to its low P_r value over a period of 40 ms.

b: Left, global $[Ca^{2+}]_i$ wave for 3Ca condition. Middle, R_f was calculated from global $[Ca^{2+}]_i$ wave using a Michaelis-Menten reaction having a maximum speed (V_m) of 800 s^{-1} and a dissociation constant (K_d) of 2 μM. R_b value was set such that the occupancies of docking site (δ = 0.3) and replacement site (ρ = 0.9) were kept constant at the resting state assuming $[Ca^{2+}]_{rest}$ of 50 nM. Right, S_f was calculated from global $[Ca^{2+}]_i$ wave using a Michaelis-Menten reaction having V_m = 60 sec^{-1} and K_d = 2 μM. S_b value was set such that the occupancy of replacement site (ρ = 0.9) was kept constant at the resting state.

c: Average $[Ca^{2+}]_i$ wave at 40-nm distance from the nearest Ca^{2+} channels for 200 Hz stimulation in 1.5 mM external $[Ca^{2+}]$ condition (1.5Ca; left). P_r was calculated from local $[Ca^{2+}]_i$ with standard allosteric model parameters (k_{on} = 5 x 10^8 $M^{-1}s^{-1}$, k_{off} = 5000 s^{-1}, b = 0.75, γ = 2800 s^{-1}, f = 31.3 ; upper middle). Expanded P_r plot displays signifcantly less widening and accumulation of late release rates compared to 3Ca (bottom middle panel; compare with A). Slow P_r was calculated from local $[Ca^{2+}]_i$ with the modified allosteric model (k_{on} = 5 x 10^7 $M^{-1}s^{-1}$, k_{off} = 2000 $M^{-1}s^{-1}$, b = 0. 5, g = 2000 s^{-1}, f = 31.3), showing a marked decrease of the slow release rate compared to 3Ca (right panel).

d: Left, global $[Ca^{2+}]_i$ wave for 1.5Ca. Middle, R_f was calculated by global $[Ca^{2+}]_i$ wave using a Michaelis-Menten reaction having a maximum speed (V_m) of 800 s^{-1} and a dissociation constant (K_d) of 2 μM. R_b value was set such that the occupancies of docking site (δ = 0.15) and replacement site (ρ = 0.9) were kept constant at the resting state assuming $[Ca^{2+}]_{rest}$ of 50 nM. Right, S_f was calculated from global $[Ca^{2+}]_i$ wave using a Michaelis-Menten reaction having V_m = 60 sec^{-1} and K_d = 2 μM. S_b value was set such that the occupancy of replacement site (ρ = 0.9) was kept constant at the resting state.

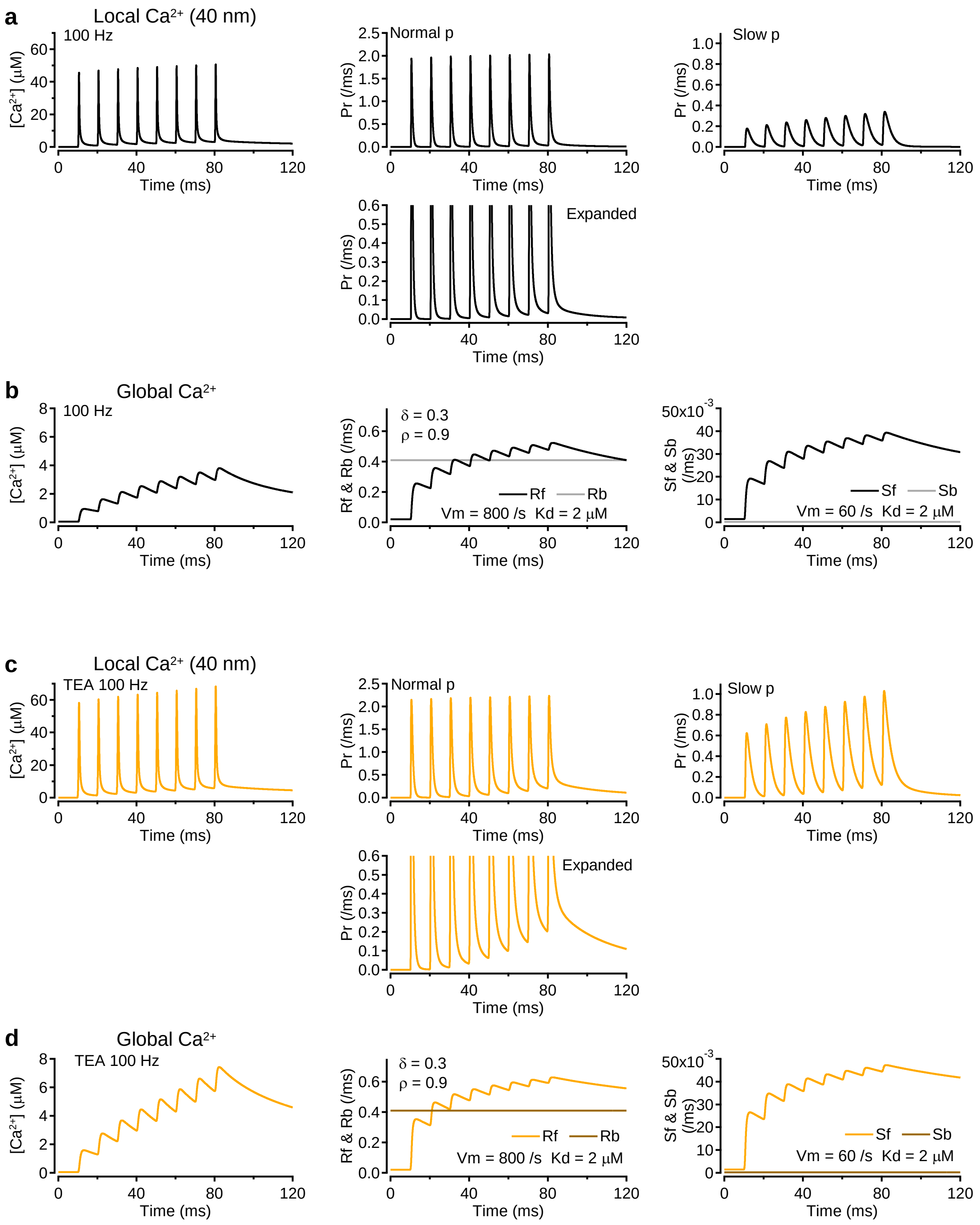

<u>Supplementary Figure 8</u><u>: Parameter values for the simulation of 100-Hz 8-pulse experiments in control and in TEA</u>

a: Average $[Ca^{2+}]_i$ wave at a 40-nm distance from the nearest Ca^{2+} channels for 100 Hz stimulation in 3 mM external $[Ca^{2+}]$ condition (3Ca; left). P_r was calculated on the basis of local $[Ca^{2+}]_i$ using the allosteric model ($k_{on} = 5 \times 10^8\ M^{-1}s^{-1}$, $k_{off} = 5000\ s^{-1}$, b = 0.75, $\gamma = 2800\ s^{-1}$, f = 31.3 ; upper middle). The bottom middle panel shows a vertically expanded plot of P_r, showing less slowing and less accumulation than at

200 Hz (compare with Supplementary Fig. 6A). As before slow P_r was calculated starting from the local $[Ca^{2+}]_i$ profile shown in the left panel, using the modified allosteric model (k_{on} = 5 x 10^7 $M^{-1}s^{-1}$, k_{off} = 2000 s^{-1}, b = 0. 5, γ = 2000 s^{-1}, f = 31.3; right).

b: Left, global $[Ca^{2+}]_i$ wave for 3Ca condition at 100 Hz. Middle, R_f was calculated from global $[Ca^{2+}]_i$ wave using a Michaelis-Menten reaction having a maximum speed (V_m) of 800 s^{-1} and a dissociation constant (K_d) of 2 μM. R_b value was set such that the occupancies of docking site (δ = 0.3) and replacement site (ρ = 0.9) were kept constant at the resting state assuming $[Ca^{2+}]_{rest}$ of 50 nM. Right, S_f was calculated from global $[Ca^{2+}]_i$ wave using a Michaelis-Menten reaction having V_m = 60 sec^{-1} and K_d = 2 μM. S_b value was set such that the occupancy of replacement site (ρ = 0.9) was kept constant at the resting state.

c: Average $[Ca^{2+}]_i$ wave at 40-nm distance from the nearest Ca^{2+} channels for 100 Hz stimulation in 3 mM external $[Ca^{2+}]$ and in the presence of 1 mM TEA (left). P_r was calculated on the basis of local $[Ca^{2+}]_i$ with standard allosteric model parameters (k_{on} = 5 x 10^8 $M^{-1}s^{-1}$, k_{off} = 5000 s^{-1}, b = 0.75, γ = 2800 s^{-1}, f = 31.3 ; upper middle). Expanded P_r plot displays significantly more widening and accumulation of late release rates compared to 3Ca (bottom middle panel; compare with A). Slow P_r was calculated by local $[Ca^{2+}]_i$ with the modified allosteric model (k_{on} = 5 x 10^7 $M^{-1}s^{-1}$, k_{off} = 2000 s^{-1}, b = 0. 5, γ = 2000 s^{-1}, f = 31.3), showing a marked increase of the slow release rate compared to 3Ca (right panel).

d: Left, global $[Ca^{2+}]_i$ wave for 3Ca plus TEA condition. Center, R_f was calculated from global $[Ca^{2+}]_i$ wave using a Michaelis-Menten reaction having a maximum speed (V_m) of 800 s^{-1} and a dissociation constant (K_d) of 2 μM. R_b was set such that the occupancies of docking site (δ = 0.3) and replacement site (ρ = 0.9) were kept constant at the resting state assuming $[Ca^{2+}]_{rest}$ of 50 nM. Right, S_f was calculated from global $[Ca^{2+}]_i$ wave using a Michaelis-Menten reaction having V_m = 60 sec^{-1} and K_d = 2 μM. S_b value was set such that the occupancy of replacement site (ρ = 0.9) was kept constant at the resting state.

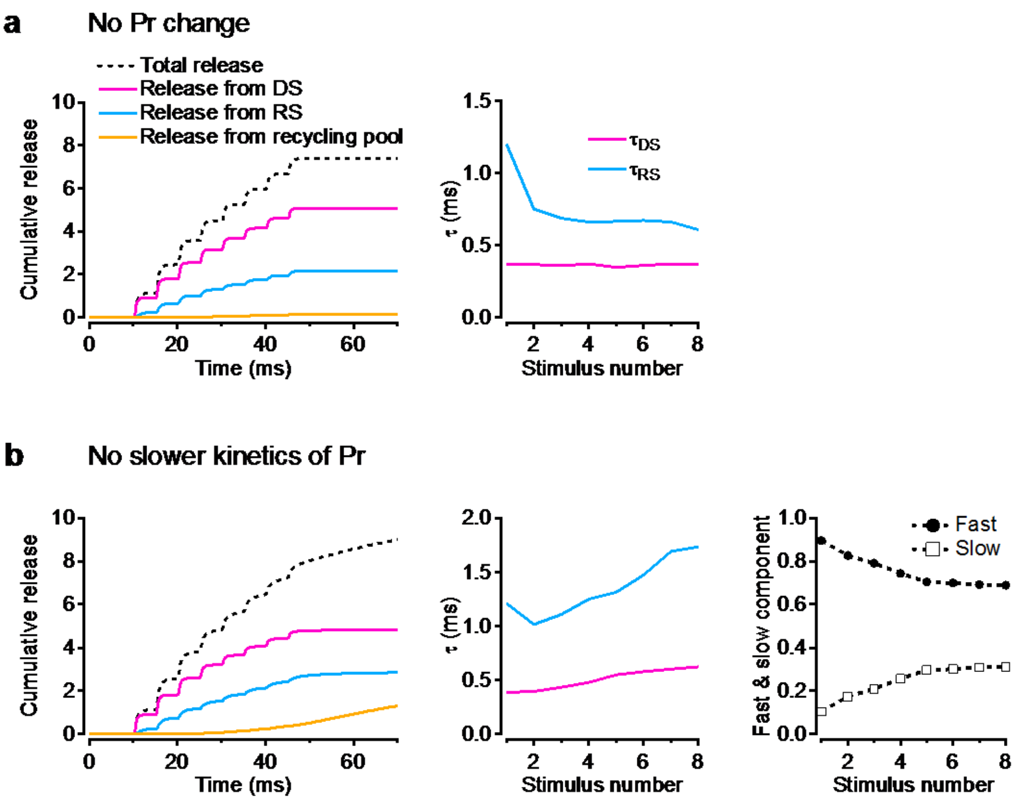

Supplementary Figure 9: Release simulations for train stimulus without changes in $[Ca^{2+}]_i$ profile or without shift to slower P_r

Kinetics of total cumulative release and cumulative release from docking site (DS), replacement site (RS), and recycling pool.

a: Monte Carlo simulation was performed using same P_r among all stimulations as that for single stimulation in 3 mM external $[Ca^{2+}]$ simulation. Left, total cumulative release and cumulative release from 3 different resources are shown: total (black), DS (red), RS (blue), and recycling pool (orange). Right, τ values for kinetics of release from DS (red) and RS (blue) were derived from fitting the cumulative release in each stimulation with a single exponential curve.

b: Monte Carlo simulation was performed without using slower P_r following previous release. Left, total cumulative release and cumulative release from 3 different resources are shown in black for total, red for DS, blue for RS, and orange for recycling pool. Middle, τ values for kinetics of release from DS (red) and RS (blue) were derived from fitting the cumulative release in each stimulation with a single exponential curve. Total cumulative release of each response was fitted with a double exponential curve having τ_{fast} of 0.49 ms and τ_{slow} of 1.87 ms, providing fast and slow component amplitudes as a function of stimulus number (right).

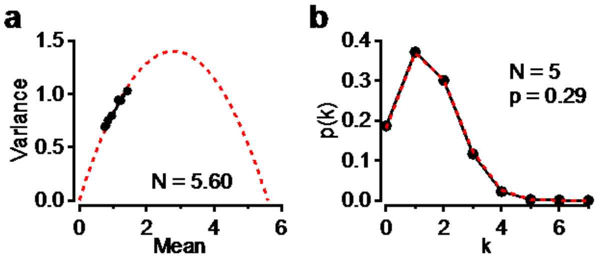

<u>Supplementary Figure 10</u><u>: Binomial statistical analysis of the number of release events in 3 mM external [Ca^{2+}] simulation</u>

a: The mean and variance of the number of events were determined in a 5-ms period after individual stimulations in 3 mM external [Ca^{2+}] simulation. The resulting curve can be fitted with a parabola indicating a release site number N = 5.60 (dashed curve).

b: The probability distribution p(k) to observe k events is displayed for the second stimulus in A. The binomial model approximates the plot from the simulation (dashed line; N = 5; mean probability P = 0.29).

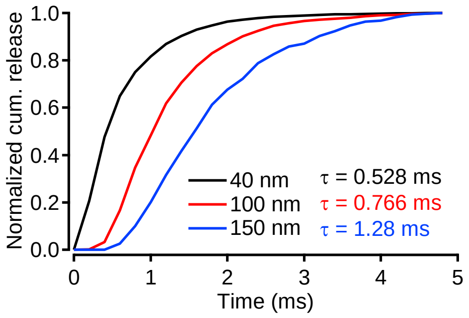

Supplementary Figure 11: Simulated latency distributions depending on the distance between release sites and Ca^{2+} channel clusters

Ca^{2+} waves at 40-, 100-, and 150-nm from the perimeter of Ca^{2+} channel cluster were used to simulate cumulative release. The cumulative release curves were normalized and fitted with a single exponential to estimate release latencies. The time constant τ was 0.528, 0.766, and 1.28 ms for 40, 100, and 150 nm, respectively.

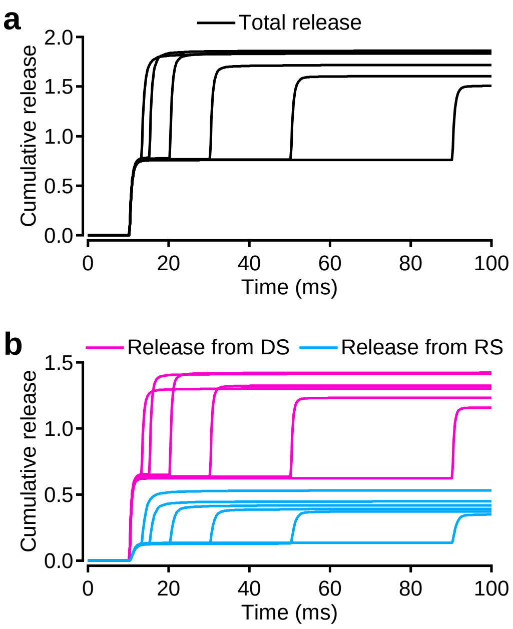

<u>**Supplementary Figure 12**: Monte Carlo simulations for paired pulse experiments</u>
a: Total cumulative release for paired stimulations with 6 different intervals.
b: Cumulative releases for SVs originating from DS and RS are shown in red and blue, respectively. This plot shows a marked increase in the relative contribution of SVs coming from RS for the second stimulation compared to the first.

Supplementary Table 1: Parameters for simulations of [Ca^{2+}] diffusion

Simulation Parameters	value	units	Reference
Simulation volume (bouton size) *x*-*y*-*z*	0.9 - 0.5 - 0.5	μm	Modified from Hillman & Chen[4]
Simulation voxel size	10	nm	
Time step for Ca simulation	0.0303	μs	
Ca^{2+} entry per single AP			
Maximal single channel current	0.2 (3 mM Ca)<br>0.14 (1.5 mM Ca)	pA	Calculated from Weber et al.[5] and Li et al.[6] for 3 mM Ca Adjusted to Ca imaging data for 1.5 mM Ca
FWHM of Ca^{2+} entry	0.34 (control)<br>0.68 (TEA)	ms	Sabatini & Regehr[7] for control. Adjusted to Ca imaging data for TEA.
Diffusion coefficient	0.22	$\mu m^2\ ms^{-1}$	Allbritton et al.[8]
Basal Ca^{2+} concentration	50	nM	Sabatini & Regehr[7]
Ca^{2+} extrusion	0.9	ms^{-1}	Helmchen et al.[9]
Endogenous fixed buffer properties			
k_{on}	200	$mM^{-1}ms^{-1}$	Sabatini & Regehr[7]
k_{off}	10	ms^{-1}	
Concentration	2	mM	
ATP calcium binding properties			
k_{on}	500	$mM^{-1}ms^{-1}$	Naraghi & Neher[10]
k_{off}	100	ms^{-1}	
Diffusion coefficient	0.22	$\mu m^2\ ms^{-1}$	
Free concentration	0.2	mM	Modified from Nakamura et al.[11]
Calretinin			
T site k_{on}	1.8	$mM^{-1}ms^{-1}$	Faas et al.[12]
T site k_{off}	0.053	ms^{-1}	
R site k_{on}	310	$mM^{-1}ms^{-1}$	
R site k_{off}	0.02	ms^{-1}	
Diffusion coefficient	0.02	$\mu m^2\ ms^{-1}$	
Concentration	0.1	mM	See Methods

Supplementary References

1. Alcami, P., Franconville, R., Llano, I. & Marty, A. Measuring the firing rate of high resistance neurons with cell-attached recording. *J. Neurosci.* **32**, 3118–3130 (2012).
2. Miki, T., Kaufmann, W., Malagon, G., Gomez, L., Tabuchi, K., Watanabe, M., Shigemoto, R., & Marty, A. Numbers of presynaptic Ca^{2+} channel clusters match those of functionally defined vesicular docking sites in single central synapses. *Proc. Nat. Acad. Sci. USA.* **114**, E5246–E5255 (2017).
3. Neher, E., & Sakaba, T. Multiple roles of calcium ions in the regulation of neurotransmitter release. *Neuron* **59**, 861–870 (2008).
4. Hillman, D. E., & Chen, S. Compensation in the number of presynaptic dense projections and synaptic vesicles in remaining parallel fibers following cerebellar lesions. *J. Neurocytol.* **14**, 673–687 (1985).
5. Weber, A. M., Wong, F. K., Tufford, A. R., Schlichter, L. C., Matveev, V., & Stanley, E. N-type Ca^{2+} channels carry the largest current: implications for nanodomains and transmitter release. *Nat. Neurosci.* **13**, 1348–1350 (2010).
6. Li, L., Bischofberger, J., & Jonas, P. Differential gating and recruitment of P/Q-, N-, and R-type Ca^{2+} channels in hippocampal mossy fiber boutons. *J. Neurosci.* **27**, 13420–13429 (2007).
7. Sabatini, B., & Regehr, W. G. Optical measurement of presynaptic calcium currents. *Biophys. J.* **74**, 1549–1583 (1998).
8. Allbritton, N. L., Meyer, T., & Stryer, L. Range of messenger action of calcium ion and inositol 1,4,5-trisphosphate. *Science* **258**, 1812–1815 (1992).
9. Helmchen, F., Borst, J. G., & Sakmann, B. Calcium dynamics associated with a single action potential in a CNS presynaptic terminal. *Biophys. J.* **72**, 1458–1471 (1997).
10. Naraghi, M., & Neher, E. Linearized buffered Ca^{2+} diffusion in microdomains and its implications for calculation of [Ca^{2+}] at the mouth of calcium channel. *J. Neurosci.* **17**, 6961–6973 (1997).
11. Nakamura, Y., Harada, H., Kamasawa, N., Matsui, K., Rothman, J. S., Shigemoto, R., Silver, R. A., DiGregorio, D. A., & Takahashi, T. Nanoscale distribution of presynaptic Ca^{2+} channels and its impact on vesicular release during development. *Neuron* **85**, 145–158 (2015).
12. Faas, G. C., Schwaller, B., Vergara, J. L., & Mody, I. Resolving the fast kinetics of cooperative binding: Ca^{2+} buffering by calretinin. *PLos Biol.* **5**, e311 (2007).
