# Supplementary material for: Two-component latency distributions indicate two-step vesicular release at simple glutamatergic synapses
Source: Nat Commun. 2018 Sep 26;9:3943. doi: 10.1038/s41467-018-06336-5 (PMC6158186; doi:10.1038/s41467-018-06336-5)
Supplement: Supplementary file 2 — Peer Review File [file 41467_2018_6336_MOESM2_ESM.pdf]

Reviewers' comments:

Reviewer #1 (Remarks to the Author):

This is an insightful study from a highly accomplished group of investigators. The authors have developed methods to measure release latencies during high frequency trains at small glutamatergic synapses with single release sites. The latency distributions exhibit multiple components that correspond to fast synchronous release, slow release as well as slower asynchronous release. The authors propose that the gradual increase in release latencies during sustained stimulation are due to ultrafast docking of vesicle from reserve and recycling pools following use of the docked vesicle pool. This is a highly plausible proposition. Moreover, based on their simulations the authors posit that there is no need to assume different Ca2+ sensitivities or distinct vesicle populations for slow or asynchronous release. Given the amount of effort this group has spent to construct these analytical tools and validate the single synapse preparation, I do not have any technical concerns. However, I think the authors should discuss and reference studies from other groups that used molecular manipulations and identified distinct Ca2+ sensors as well as vesicle populations for the slower forms of release. There appears to be multiple mechanisms that can give rise to asynchronous release and some synapses even predominantly rely on these slower forms of release. Therefore, although simulations may not uncover a “need” for molecular diversity, there, nevertheless, seems to be significant divergence of molecular mechanisms.

Reviewer #2 (Remarks to the Author):

The paper by Miki et al. examines the time course of release during high-frequency trains at small glutamatergic synapses. To address this question, the authors use electrophysiology, calcium imaging, and modeling. The main findings are:

- Synaptic latency exhibits a single fast component at train onset, but a slow component of increasing amplitude during train stimulation.
- The contribution of the slow component increases with stimulation frequency and with release probability, but decreases when blocking docking with latrunculin.
- Modeling suggests that the slow release component is generated by a two-step process, with docking and release in immediate succession.

Based on these results, the authors conclude that the time course of release is less constant, and that docking and release are more integrated than previously thought.

Overall, this is a nice paper. The conclusions are interesting and important, the experiments are performed adequately, the analysis is nicely quantitative, and the manuscript is generally well written. However, it is also clear that major revisions are required before this manuscript can be published.

Major points:

1. A major assumption of the model is that the docking site occupancy of 0.3. However, it is unclear how the authors arrived at this value. Notably, the number is significantly lower than previous estimates from the same group at GABAergic synapses (0.51 to 0.84, Pulido et al., 2014). Was the number constrained by the variance–mean data in Supplementary Fig. 8? If so, these data should receive more emphasis. Finally, how the replacement site occupancy was determined is also unclear.

2. Another key assumption of the model is that Rf is Ca2+ dependent. What was in the end the hard evidence for this? At the very least, this point should be better discussed.

3. The covariance analysis is also fuzzy. First, it remains unclear which parameter was analyzed in the covariance calculation. The authors state "release counts", but this is not very clear. Second, the fact that there is a negative correlation does not mean that the vesicles are released by the same vesicle pools and release mechanisms. Partial overlap could be sufficient to generate such a correlation.

4. Asynchronous release is not very well quantified. Both the relative amount of asynchronous release and the decay time course should be analyzed. Vague statements such as "super slow component" should be replaced by quantitative data.

5. As the authors know, several issues are apparent with the deconvolution method used to extract the time course of release. First, data were probably filtered, which may limit the temporal resolution. This is never mentioned in the paper. Second, miniature EPSCs are used for deconvolution, which originate in different populations of synapses. Third, the deconvolution analysis requires linearity of the postsynaptic reporter. Apparently cyclothiazide or kynurenic acid were not used in the present study. Additionally, voltage clamp conditions in the experiments (e.g. postsynaptic access resistance) are insufficiently described. Finally, how exactly was deconvolution mathematically performed?

6. Recordings were performed at very young animals, P12 - P16. Whether the conclusions apply to more mature synapses remains unclear. Control experiments in more mature synapses should be performed to document the presence of the slow release component under these conditions.

7. The presentation of the data requires improvement. I gave several examples below, but work is needed beyond these suggestions. I also would like to see more original traces in the first figure, showing individual early and late release events rather than secondary and tertiary data. Finally, the synaptic properties should be briefly discussed in relation to cerebellar granule cell activity in vivo (Margrie lab, Jörntell lab).

Minor points:
Line 23: The type of synapse examined should be mentioned in the title and the abstract.

Line 54: I don't see any time course of release data in Ref. 11.

Line 57: "Giant synapses … can solve this problem " – unclear.

Line 62: Tight coupling between Ca2+ channels and release sensors at some synapses versus loose coupling at other synapses (Vyleta et al., 2014, Science) may be another reason to assume that the time course of release is not constant. This should perhaps be mentioned.

Line 73: "synaptic fatigue" should be replaced by more precise terminology.

Line 86: "have only been obtained at multiple synapses" - the statement may be misleading, since Sherrington (who coined the term) defined the synapse as the sum of all contacts / active zones between two neurons.

Line 97: "alternatively be considered as the time course of release rate" - this unclear statement should be replaced.

Line 99: Precise values of decay time constant (and probably half-duration) should be given (mean +- SEM).

Line 101: "0.3/0.2 ms" - unclear.

Line 150: The role of global calcium is overemphasized, here and elsewhere in the paper. Clearly, there are multiple factors contributing to asynchronous release and facilitation.

Line 170: I know it is difficult, but it would be nice to have statistics on the statement that the first response is monoexponential, whereas the second requires two exponential components.

Line 221: Again, precise numbers need to be given.

Line 227: Using a constrained fit for this important conclusion is problematic. The authors should show that results from a free unconstrained fit are at least consistent with the conclusions.

Line 239: "Anti-actin drug" – more precise statements are necessary.

Line 245: n = 3, n = 5 - precise intersection points should be given.

Line 265: The TEA concentration should be specified in the text, not only in the legend. Likewise, the latrunculin concentration should be given.

Line 269: Again, precise values (mean +- sem) should be given.

Line 337: "By contrast no correlation was found" – it is unclear how this fits to the hypothesis that synchronous and asynchronous release share the same release sites / pathways. The decay time constant for asynchronous release is > 100 ms at some

synapses.

Line 352: The Monte Carlo simulations have to be better explained in the Methods.

Line 357: "Rate limiting for a rested synapse …" – talking about rate liming steps in an equilibrium condition seems confusing.

Line 363: "confirming" – "consistent with", at best.

Line 432: Recent results (Kawaguchi & Sakaba, 2017, Cell reports) were apparently more consistent with 100 nm coupling distance at parallel fiber synapses. This should be mentioned.

Line 447: "Tau slow component may be a hallmark of synapses specialized in very fast signaling" - this is true for the calyx, right?

Line 496: Didn't Murthy and Stevens, 1999, Nature Neuroscience first obtain evidence for undocking? They should probably be cited here.

Line 499: "1st option" – what is first, what is second?

Line 547: I know, it is difficult, but it would be nice to have a statement whether the authors think they primarily recorded from basket cells, stellate cells, or a mixed population.

Line 573: Inclusion criteria have to be specified much more quantitatively.

Line 603: Why was the membrane potential held at such negative values?

Line 610: A proper description of analysis methods is missing from the paper. For example, it would be clearly important to know how data were processed for deconvolution (see above).

Line 631: What is meant by "significantly different" (which should be reserved for statistical comparisons)? Maximal deviations should be given instead.

Line 634: As P/Q type channels contribute to release at this synapse, Li et al., 2007, Journal of Neuroscience would be a better reference.

Line 635: Sabatini and Regehr, 1997, report a peak open probability much smaller than 0.6. Thus, both Borst and Sakmann, 1998, J Physiol and Bischofberger et al., 2002, J Neuroscience would be more appropriate references.

Line 655: The stability criterion should be better explained, and the corresponding time steps should be specified.

Line 669: The deviations from the previously published allosteric release model should be better explained (e.g. koff of 4000 s-1, maximal release rate of 6000 s-1). Furthermore, the Lou et al., 2005 model does not use gamma, but rather l+ as a release rate. Finally, what is gamma (g)?

Line 702: “unpublished results …” – more details need to be given.

Line 946: “m” should be “mean”.

Line 986: These are dashed, rather than dotted lines.

Line 987: “Transition” is misspelled.

Figure 1A: These traces do not look like individual traces, in contrast to what is claimed in the legend.

Figure 1F: Model parameters should be given in proper scientific notation (including units) or deleted. See also in other figures.

Figure 3: The panel labeling is chaotic.

Supplementary Fig. 1: On which original SDS-FRL data were the Ca2+ channel distributions based? This should be stated more clearly.

Supplementary Figure 2: The authors should state more clearly how the absolute Ca2+ concentrations were calculated. They used a nonratiometric indicator dye, so this is not trivial.

Legend Supplementary Fig. 3: “would” is misspelled.

Figures throughout: I find it confusing that the release rates are given per 0.2 ms bin. They could be easily given in standard units (s-1 or ms-1).

Supplementary table 1: “Regehr” is misspelled several times.

Reviewer #3 (Remarks to the Author):

Two-component latency distributions indicate two-step vesicular release at simple glutamatergic synapses
Miki , Nakamura, Malagon, Neher and Marty

The Marty lab has championed synapses between parallel fibers and molecular layer interneurons (stellate or basket cells) as model excitatory “simple synapses”, that is, a

preparation capable of recording responses from single active zones. The advantage of a simple synapse is that the complete history of synaptic activity can be documented. One can then measure parameters such as the size of the readily releasable pool, refilling speeds, synaptic delay and postsynaptic receptor saturation and desensitization. In previous manuscripts, the Marty lab has demonstrated that multi-vesicular release occurs at these active zones, demonstrating that there must be multiple docking sites. With the Shigemoto lab, they observed three clusters of calcium channels each comprised of nine channels. Modeling of the responses suggested that there are four docking sites with a roughly 50% occupancy, a 70% probability of release. The 'two-step' model identifies a second replacement pool which can rapidly refill the docking sites within the 5 ms between stimulations.

What is not known is whether docked vesicles and replacement vesicles are poised in different states. In this manuscript the authors use high frequency stimulation to stress vesicle replenishment rates so that latencies of vesicle fusions reflect rates of docking. They find that there are two vesicle pools: fast fusion and slow fusion vesicles. Their model claims that docked vesicles are poised for rapid fusion with calcium influx, but that newly replenished vesicles exhibit a slower release rate in response to calcium influx. They then incorporate their data into a comprehensive model, combining data from calcium clusters, calcium imaging and the two-step docking-site refilling model from previous publications.

In this manuscript the authors demonstrate that at small synapses, trains of action potentials result in a broadening of quantal latencies. These results are fit into a model demonstrating:

1. Fast latencies are derived from fully docked vesicles.
2. Slow latencies are derived from vesicles transiting from the replacement pool directly to fusion.
3. Refilling of the pools is calcium dependent.
4. Refilling of the pools is dependent on the actin cytoskeleton.

The electrophysiological results can be fit into a two-step model for vesicle release. They suggest that these steps are sequential and might correspond to a synaptic vesicle transiting all the way through docking to fusion. As the synapse is depleted, the docked vesicles are consumed, and further quanta during high-frequency stimulation arises from newly arrived vesicles transiting directly through to fusion without a stable docking state (these later events are the slower component). Finally, they show that the fast component, the slow component, and asynchronous release are all interdependent. This finding makes it likely that all of the components result from a common pool of vesicle utilizing a single, common release machinery. In closing this is a fascinating paper, and makes me wonder what these parameters are at facilitating synapses. Is the docked pool essentially empty, and all fusions from the replacement pool?

In conclusion, the data are convincing, the model is thought-provoking, and the manuscript important. My critique is largely restricted to issues of clarification.

1. The authors suggest that there are docked vesicles and a replacement pool poised to refill the empty docking sites in Figure 4A and in previous manuscripts. As the authors point out, "Given the very fast kinetics of 2-step release, where docking and release occur within ~2 ms, it seems likely that SVs are already engaged with the release apparatus when they are located at the replacement site." Thus the replacemnt pool does not engage emptied "docking" sites but rather it seems that vesicles engaged with the release machinery are bouncing between a fast release state and a disengaged slow state.

2. Although the authors explicitly state that vesicles must pass through these two steps, it is not clear to me why this could not be satisfied by twice as many docking sites in which half of the vesicles are not in a readily releasable state.

3. Are there morphologically docked vesicles at distance from the active zone? Could the increase of calcium recruit more distant vesicles at late points in the stimulus train? I know this was dealt with in the previous paper, but a sentence addressing this would be helpful.

4. This is a dense manuscript, which will be hard for anyone but experts to navigate. In the Introduction, the manuscript is devoid of descriptions of what is happening. The authors should briefly mention the components in synaptic delay, specifically, the action potential invading the bouton, the speed of calcium channel opening, the increased driving force during the falling phase of the action potential, calcium binding to synaptotagmin, SNARE-mediated fusion, and neurotransmitter diffusion, and activation of ligand-gated ion channels. These events define the lag phase before fusion. The authors should also define 'latency' operationally. (I assume it is action potential peak to the intersect of the rise of postsynaptic current).

5. Latrunculin causes the cross-over point for fast versus slow release to move from the 3rd to the 6th stimulus – a lag of 15 ms. I don't think that this necessarily suggests a dependence on actin. It seems like an indirect effect is more likely, for example a block in vesicle clearance by endocytosis seems more likely.

Minor considerations:

Pg. 1. "The latency distribution exhibits a single fast component at train onset but later reveals a slower component with increasing amplitudes"

"Increasing amplitudes" is too vague a description. I initially read this as referring to increases in quantal content.

Figure 1A: Four traces are shown, nicely illustrating events in which 0, 1 or 2 vesicles fused. It is difficult to separate these out visually. Can the authors improve the figure? Inverting the order of the traces would help associate the traces, so that the EPSC is on the bottom, the deconvolution above that, and the latencies on top would reduce the space that separates the EPSC and the deconvolution in the current figure. Also adding a label "0", "1" or "2" would help associate the traces.

Pg. 3. Please state temperature when describing recording conditions.

Pg. 3, L 115-118. Is the value for global calcium measured or modeled? [Fig 1D; Supp Fig 2].

Pg. 3. L. 123: "obtained using the red profile in Fig. 1D together with an allosteric release model."
It might be clearer if the authors stated "and allosteric release model for calcium-mediated release and the calcium curve at 40 nm (Fig 1D)."
I was convinced there was a typo, since I was still staring at panel 1E.

Pg. 4. L. 147: "AR" for "asynchronous release" is an unnecessary acronym.

Pg. 6. "Meanwhile the reduction in the τfast component with i was less rapid in the lower external [Ca2+] due to a reduction of synaptic depression of this component, leading to a slower decrease of the τfast component in 1.5 mM external [Ca2+] compared to 3 mM."

Sometimes the nomenclature leads to awkward sentences. It would be more clear to write the sentence as: "Meanwhile the reduction in the τfast component during the stimulus train was less rapid in the…"

Pg. 6. "(Note that the larger value of τslow in Fig. 2 results from a lack of consideration of AR in that figure as well as from a longer time window for fitting.)"

I understand the explanation; I don't understand the logic of why the change in analysis was made. Can the slow component not be seen in paired pulses if asynchronous release and a longer time window are not used? I realize that in these experiments the slow component is only a tiny fraction of the response – but are the authors forcing its observation by altering the parameters until something that looks like the slow component appears. If the asynchronous component is removed from the analysis of Fig. 2 can the slow component still be seen? Just wondering.
Pg. 10. "A second mechanism, increasing Ca2+ entry, also conflicts with experimental data (Suppl. Fig. 2)."

This statement needs further explanation. I assume you mean that in individual APs the calcium influx remains constant. Thus, the slow component is not due to lengthening of the calcium influx. However, Suppl. Fig. 2 also dramatically illustrates that the calcium buffering capabilities of the neuron do not keep up with the AP train. During the stimulus train the calcium increases linearly at both 3mM and 1.5mM calcium – with no indication of leveling off.

Pg. 12. "A recent electron microscopy study using flash-and-freeze technique revealed that in certain conditions the number of SVs located 0-5 nm away from the presynaptic AZs is transiently increased 10 ms after a presynaptic AP, reversing with a time course similar to that of paired pulse facilitation."

I think you need to be explicit in describing what certain conditions means. In this case the result was seen in C2B mutants in synaptotagmin. Whether these same results would hold in wild-type synapses is currently unknown.

General:
We would like to thank all three reviewers for insightful and constructive comments on our ms. In the following, we explain how we have dealt with the various points raised by the reviewers.

Several changes have been introduced in the figures, as follows.

Old Figure 3 has been split into two figures, new Figures 3 and 4. This change was triggered by the (justified) remark by Reviewer 2 that in the old Figure 3, panel labeling was 'chaotic'. Trying to fix this issue has led to the proposed separation in two figures. Given the size and complexity of old Figure 3 we trust that splitting this figure in two makes reading easier.

We have added a new supplementary figure to contrast the small jitter for presynaptic APs with the much larger jitter of EPSC onset. This figure serves as (partial) justification to study the calcium-SV release coupling as the main source of synaptic jitter. It was inspired by Major comment 4 of Reviewer 3.

We have added another supplementary figure to illustrate latencies during a 200 Hz AP train for an older preparation (4 weeks old rat). This new figure was added in view of Major point 6 of Reviewer 2.

Finally in Discussion, the section dealing with the comparison between our results and those in the calyx of Held has been revised.

Reviewer 1:

We agree with this Reviewer that our covering of earlier literature on some issues was shallow, including molecular studies, the link between delays and SV-VGCC separation, and asynchronous release. In the corrected version we have addressed these issues as follows:
In the Introduction, we have added two paragraphs dealing with the effects of varying SV-VGCC separation on synaptic delays, and with earlier studies on asynchronous release.
At the end of Discussion, we have introduced a new section linking our findings with earlier molecular studies of synaptic proteins. Because of the vast extension of the relevant literature, we have chosen to focus on a single synaptic protein, namely synaptotagmin 7. With this example, we try to show how our findings may contribute to the interpretation of knock-out studies.

Reviewer 2:

Major point 1:
Docking site occupancy at rest is almost certainly synapse specific, so that it is not surprising that our estimate at PF-MLI synapses is different from that at MLI-MLI synapses. Already in our previous report (Miki et al., 2016), we found a lower occupancy (0.45) than that of GABAergic synapses. In addition to the difference between preparations, there is also a methodological difference that needs to be taken into account. The previous model (Miki et al., 2016) was a

simpler model than the present model, as we prohibited release of newly recruited vesicles. This difference of model accounts for the lower occupancy value found in the present study (0.30 instead of 0.45). As before, the optimal occupancy was found by an optimization of the entire fitting procedure. We have clarified these points both on p. 4 and in a new paragraph in Methods, p. 20. Regarding the occupancy of replacement site, our previous report indicated an occupancy of 1. In the present version of the model, replenishment of the replacement site is reversible. Therefore, the occupancy value at resting state results from an equilibrium, so that we set the occupancy slightly below 1, at 0.9.

Major point 2:
We do not have any direct evidence from our data concerning the calcium dependence of Rf. However, the evidence from the literature indicating that SV replenishment is calcium-dependent is overwhelming. Since Rf is the main SV replenishment step, it is very likely calcium dependent. Also our previous interpretation of facilitation being due to SV replenishment necessitates that Rf is calcium dependent (Miki et al., 2016). We have now added text on p. 5 to clarify these points.

Major point 3:
A first point of the Reviewer is that the description of covariance analysis was lacking in our original ms. To cover this point we have now included a new paragraph in Methods (p. 18).

A second point concerns the interpretation of covariance results. Our finding of a negative correlation in Fig. 4 indicates an interaction between the pathways leading to synchronous and asynchronous release. In particular this finding is difficult to reconcile with the widespread assumption that asynchronous release occurs at release sites that are separate from those of synchronous release. Slow supply of separate pathways by a common pool of reserve SVs would provide some coupling between the two processes, as suggested by the Reviewer, but this coupling would be unlikely to produce enough negative covariance to account for the results. On the other hand, comparison between experimental results and simulations in old Fig. 3I (now Fig. 4g) show that the 2-step release model predicts an amount of negative correlation that is within a factor of 2 of that observed both in 3 Ca, and in 3 Ca + TEA. To stress these important findings we have introduced a short explanation of the logic underlying covariance analysis (p. 10), and we have expanded the description of simulations in Fig. 4g. See also further related comments below ('line 337').

Major point 4:
To address this point, we have quantified asynchronous release in various conditions, giving mean values and sem based on individual experiments (pp. 7-8). We have also included statistical comparisons, for example comparing asynchronous release in control and in LatB. Finally we have as requested removed the vague 'super slow component' statement.

Major point 5:

We agree that the deconvolution method was described too succinctly in the original submission. The deconvolution method is described and evaluated in detail in an earlier publication (Malagon et al., 2016). We have added a new par. in Methods to recall the main features of this analysis ('Decomposition of EPSCs', pp. 16-17).

Recording bandwidth is limited on one side by the combination of series resistance and input capacitance (that together define a time constant of about 100 microseconds), and on the other side by dendritic filtering (similar time constant, see Pouzat and Marty, 1999). We have extensively studied the time resolution of our deconvolution (the minimum separation of two consecutive EPSCs that are distinguished by our analysis) and found that depending on the experiment, it ranges between 0.2 and 0.3 ms (Malagon et al., 2016, Fig. 2). This corresponds to the bracket 0.2-0.3 ms indicated in our present work. These points are now explained in the new paragraph 'Decomposition of EPSCs', pp. 16-17).

'Miniature EPSCs are used for deconvolution, which originate in different populations of synapses.' In fact, we use for deconvolution quantal events occurring during asynchronous release (Malagon et al., 2016). As shown in this paper, these events are synapse specific, and they originate entirely, or almost entirely, in the simple synapse that is under study. Therefore, the miniature EPSC waveform used for deconvolution has the same time course and the same amplitude as those of evoked quantal events. In order to clarify this point, we have now expanded the part of Methods dealing with the criteria for identification of simple synapses (p. 16).

'The deconvolution analysis requires linearity of the postynaptic reporter.' True but again, this has been analysed at length in the earlier Malagon et al. paper. In that paper we show that the amplitude of quantal events decreases at short separations due to the unavailability of previously activated receptors for renewed activation. Unlike standard studies using EPSC peak amplitudes, our analysis almost fully compensates for receptor activation (apart from the 0.2-0.3 ms long blind period), and reports unbiased numbers of released SVs. Finally a correction procedure is used to correct for undetected events in the blind 0.2-0.3 ms period, as explained in Malagon et al., 2016. These points are now made in the new section 'Decomposition of EPSCs, pp. 16-17.

'How exactly was deconvolution mathematically performed?' This is described in Malagon et al., 2016.

Major point 6:
To address this point, we have incorporated new data from 4 week old animals. When analyzing these data we found that latencies display the same components as at 2 weeks: fast, slow and asynchronous release. This is now reported in a new paragraph of Results (p. 7), and is illustrated as a new supplementary figure (Suppl. Fig. 6).

Major point 7:

'More original traces in the first figure': To cover this point we have redone Fig. 1A, selecting a new set of data, and improving the presentation. We trust that this figure is now easier to understand. We keep this figure focused on the first stimulus in a train for the sake of simplicity. More complete original data including late events can be found in earlier publications (Malagon et al., 2016; Miki et al., 2016, 2017).
'Relation to cerebellar granule cell activity in vivo': To cover this point we have incorporated a new paragraph in Discussion (p. 13).

Minor points:
Line 23: As suggested we have now indicated the synapse type in the Abstract. We have not added this information in the title though. This is the first study describing release latencies at simple glutamatergic synapses. Given the very basic aspect of the study, it is possible, and even likely, that the results will apply generally to this type of small synapses. We do not want to give the impression that our results are specific of a certain type of synapses when the opposite is likely to be true.

Line 54: We have changed our phrasing to cover this point.

Line 57: We agree that the text was not clear, and we have removed this text altogether.

Line 62: Done; we have added a paragraph covering this point.

Line 73: Done.

Line 86: Done.

Line 97: Done.

Line 99: As suggested we are now giving a mean and a confidence interval for the decay time constant of the latency distribution (p. 3).

Line 101: Done

Line 150: The Reviewer is right: focusing on global calcium is an oversimplification. However the emphasis on global calcium does not come from us, it comes from the literature, starting with the 'residual calcium hypothesis' originally developed at the neuromuscular junction. As discussed in the legend to Supplementary Figure 4, there is another reason to focus on global calcium, namely that this leads to a simpler treatment of the simulation, implicating less free parameters, than an alternative treatment that would be based on more local calcium.

Line 170:
To address this point, we have performed the Bayesian Information Criterion (BIC) of single exponential vs. double exponential fits for the responses to a single AP or to the 2nd AP of a pair. Whereas in the first case (single AP), the

statistical score is smaller for single than for double exponential fits, in the second case (2nd AP of a pair), the score are markedly better when using a double exponential fit. This analysis has been included in the presentation of the results of Fig. 2 (pp. 5-6). It supports our proposal that the slow component is much more prominent with two APs than with one.

Line 221: To address this point we have revised our quantitative analysis and given more precise numbers (p. 7).

Line 227: In view of the reviewer's comment we have now done unconstrained fits. Tau fast and slow from unconstrained fits are shown below.
LatB: τ_{fast} = 0.38 ms, τ_{slow} = 1.81 ms
3Ca: τ_{fast} = 0.49 ms, τ_{slow} = 1.43 ms
1.5Ca: τ_{fast} = 0.48 ms, τ_{slow} = 2.06 ms
3Ca/100Hz: τ_{fast} = 0.44 ms, τ_{slow} = 1.97 ms
3Ca/TEA/100Hz: τ_{fast} = 0.59 ms, τ_{slow} =2.93 ms
These results show that tau values are similar for all conditions. Therefore we fixed tau values of 0.49 and 1.87 ms in order to compare the changes of fast and slow components in all conditions.
In order to document the unconstrained fit we now give in the text the ranges for τ_{fast} and τ_{slow} across experimental conditions (p. 7).

Line 239: Done.

Line 245: Corrected to i = 3, i = 6 in ms.

Line 265: Done.

Line 269: As suggested we now provide confidence intervals for both τ_{fast} and τ_{slow} (p. 7).

Line 337: The lack of crosscorrelation for the 0-5 vs. 35-65 ms time periods is obtained both experimentally and in simulations, as illustrated in old Fig. 3I (new Fig. 4g). Altogether, our model correctly predicts both the amplitude and the kinetics of the correlation. Concerning the duration of asynchronous release: According to our simulations it is the time course of return of the global calcium concentration that limits the duration of asynchronous release in our preparation. (See also our response to Major point 3 above)

Line 352: Done.

Line 357: Text has been corrected.

Line 363: Corrected.

Line 432: We now quote the 100 nm value by Kawaguchi and Sakaba when we report our 40 nm estimate. The 100 nm value is not consistent with previous findings on PF-MLI synapses in cerebellar slices -to start with, AZs in slices are too small to accommodate such a value. There are many differences between

cultured preparations and their in vivo/slice counterparts, including the size of presynaptic varicosities. The difference between SV-VGCC distance estimates in our study vs. Kawaguchi and Sakaba is therefore not surprising.

Line 447: The reviewer is right, our argument was misleading. Text has been corrected (p. 13). In the new version, we explain that a high Rf arises in sensory sytems dealing with fast bursts of relatively short duration, like the PF-MLI synapse. On the other hand, the hearing pathway must keep track of the precise timing of individual APs and this is done with many sites functioning in parallel (in calyx synapses).

Line 496: Done.

Line 499: Corrected.

Line 547: We have used a mixed population, like in our previous studies. A comment to this effect has been included in Recording Procedure in Methods (p. 16).

Line 573: Done (new text p. 16).

Line 603: We found that holding the soma at negative potentials (around -90 mV) helped to keep stable Ca responses during long Ca imaging. This is now explained in Methods (p. 17).

Line 610: Done (new text p. 16-18).

Line 631: Done. We have expanded this analysis by adding new panels in Suppl. Fig. 2, and we have changed the text on p. 18 accordingly.

Line 634: Done.

Line 635: Done.

Line 655: The time step is 0.03µs. We have now incorporated this information in Methods (p. 18).

Line 669: gamma is the release rate for the 5th site in the allosteric model. It is identical to l_+ x f^5. In Methods, we added a sentence to explain this point (p. 20). We found a better fit when using our parameter values in PF-MLI synapse than when using those of the Lou et al. paper as shown in Fig. 1.

Line 702: Done.

Line 946: Done.

Line 986: Done.

Line 987: Done.

Fig. 1A: Indeed black traces represent individual traces. One key advantage of the preparation is the very large size of quantal EPSCs, and the small size of the cell, giving altogether an excellent signal to noise ratio. In any case, we have redone this panel to make it easier to understand.

Figure 1F: We removed model parameters from this figure panel to improve clarity.

Fig. 3: The Reviewer is right to point out that this figure was not satisfactory. We have now split it in two new figures, Fig. 3 and Fig. 4.

Supplementary Fig. 1 (Suppl. Fig. 2 in the present version): Done.

Supplementary Fig. 2 (Suppl. Fig. 3 in the present version): We added a new paragraph in Methods to explain the calculation of Ca concentration from Ca imaging data (p. 18).

Legend Supplementary Fig. 3: Corrected.

Figures throughout: Done.

Supplementary table 1: Corrected.

Reviewer 3:

Major Point 1:
The Reviewer is quite right, the exact nature of what we have called 'replacement site' is still uncertain. In our model, occupancy of the replacement site is compatible with occupancy of the docking site. This feature is necessary to explain our previous analysis of cumulated counts of SV release (Miki et al., 2016). In another view however, which is advocated in the very recent Chang et al. paper, the distance between the two sites may be so short (<10 nm) that simultaneous occupancy is not possible. In that case the two sites may be two states of docking , e.g. relatively loosely connected and tightly docked. While the two models are distinct and predict different outcomes, they are sufficiently close to each other to contemplate the possibility that they may both apply, depending on differences in preparations and in experimental conditions. We have added a new sentence in Discussion (p. 14) to stress the remaining uncertainty concerning the distinction between docked and replacement SVs.

Major Point 2:
A parallel model with twice the number of docking sites cannot be entirely excluded but is very unlikely. Such a model would severely distort the 'N1 parabola' obtained in variance-mean analysis of SV counts unless highly improbable changes of release probability are assumed during a train; furthermore this model is difficult to reconcile with latrunculin/blebbistatin data

(see detailed discussion of these points in Miki et al., 2016, notably Figure S7 and its figure legend).

Major Point 3:
To clarify this point, we have added two sentences in the Discussion (p. 12). In the Calyx of Held it was indeed postulated that less favourably located vesicles contribute to exocytosis. This, however, is restricted to very strong stimulation (longlasting depolarizations or release of Ca from caged-Ca), with such vesicles contributing only very little to release during AP-trains (Sakaba, 2006; ref. 60). At PF-MLI synapses a contribution of distant SVs seems even less likely. Given the compact arrangement of the AZ at these synapses the more distant SVs would need to fuse outside the AZs. But the possibility that SVs could dock and fuse away from the AZ would be difficult to reconcile with the existing literature. SVs are supposed to dock by binding with a protein complex including RIM. These proteins are in the AZ and only in the AZ. In our cryofracture EM paper (Miki et al., 2017), the AZ outline was based on a cocktail of antibodies to AZ proteins including RIM. In the recent Blanpied paper on nanocolumns (Tang et al., 2016), exocytosis is colocalised with RIM spots. Furthermore, in the old Heuser-Reese papers, exocytosis remains constrained to the AZ, while endocytosis is seen on the side of the AZ.

Major Point 4:
To address this point, we have added a new paragraph in Results, together with the description of Fig. 1, along the lines suggested by the Reviewer. We have also added a new supplementary figure (Suppl. Fig. 1 in the revised version) to illustrate the lack of AP latency jitter in paired experiments.

Major Point 5:
The 15 ms lag gives a false impression on the time of action of the target of latrunculin. When analysing the responses to individual APs, it appears that latrunculin potently suppresses the second component of release with time constant around 2 ms. This is clearly visible already for the 2nd AP, 5 ms into the train. By contrast the time constant of ultrafast endocytosis has been estimated at 50 ms or longer (Watanabe et al., 2013). Likewise, 'site-clearing' has been shown at the Calyx of Held to become limiting only for very strong stimulation (longlasting depolarizations or long trains of stimulation at high frequencies). Based on these numbers it does not appear realistic to attribute the effects of latrunculin to a block of endocytosis.

Minor:

Pg. 1: Fixed.

Fig. 1A: Fixed.

Pg. 3, l. 118: We have modified the text to clarify what is calculated and what is measured. Also we have explained the correction for added buffer in Suppl. Fig. 3 (old Suppl. Fig. 2).

Pg. 3, l. 123: Fixed.

Pg. 4, l. 147: Fixed.

Pg. 6: "Meanwhile…" Fixed.

Pg. 6: "(Note that the larger value…)" For the analysis of Fig. 3, a time dependent asynchronous release curve was calculated as explained in Methods (new para. p. 18). The determination of τ_{slow} was performed after subtraction of this time dependent curve, as illustrated by the lower panel of Fig. 3b. By contrast in Fig. 2, the amplitude of asynchronous release was so small that obtaining such a curve was impractical. Therefore in Fig. 2, the biexponential fit was performed on the initial data, not after subtraction of asychronous release. In addition as stated, the restriction of the data to 5 ms time window introduced an avoidable bias when fitting the train data, effectively minimizing the contribution of the slow phasic component.
As the slow component has a time constant of 6 ms in Fig. 2, we cannot call it asynchronous release (asynchronous release being attributed to processes lasting at least 10 ms: see Kaeser and Regehr, 2014).

Pg. 10: This is correct: The peak calcium increases almost linearly with AP number. This finding is consistent with our assumption of a large concentration (2 mM) of a low affinity (50 µM) intrinsic buffer (Suppl. Table 1), as well as with our simulations (Suppl. Fig. 7b, left; Suppl. Fig. 8b, left).

Pg. 12: Fixed.

REVIEWERS' COMMENTS:

Reviewer #1 (Remarks to the Author):

The authors have addressed my earlier comments. I don't see any issues that would preclude publication.

Reviewer #2 (Remarks to the Author):

The authors have carefully addressed my comments (as of course expected from this group) and revised the paper. From my point of view, this manuscript is suitable for publication without any further changes.

Reviewer #3 (Remarks to the Author):

The authors have satisfactorily amended the manuscript.
